# Bioenergetic-related gene expression in the hippocampus predicts internalizing vs. externalizing behavior in an animal model of temperament

**DOI:** 10.3389/fnmol.2025.1469467

**Published:** 2025-03-04

**Authors:** Elaine K. Hebda-Bauer, Megan H. Hagenauer, Daniel B. Munro, Peter Blandino, Fan Meng, Keiko Arakawa, John D. H. Stead, Apurva S. Chitre, A. Bilge Ozel, Pejman Mohammadi, Stanley J. Watson, Shelly B. Flagel, Jun Li, Abraham A. Palmer, Huda Akil

**Affiliations:** 1 Michigan Neuroscience Institute, University of Michigan, Ann Arbor, MI, United States; 2 Department of Psychiatry, University of California San Diego, La Jolla, CA, United States; 3 Seattle Children’s Research Institute, University of Washington, Seattle, WA, United States; 4 Department of Neuroscience, Carleton University, Ottawa, ON, Canada; 5 Department of Human Genetics, University of Michigan, Ann Arbor, MI, United States; 6 Department of Pediatrics, University of Washington School of Medicine, Seattle, WA, United States; 7 Institute for Genomic Medicine, University of California San Diego, La Jolla, CA, United States

**Keywords:** temperament, hippocampus, RNA-Seq, locomotor activity, anxiety, energy metabolism, eQTL

## Abstract

Externalizing and internalizing behavioral tendencies underlie many psychiatric and substance use disorders. These tendencies are associated with differences in temperament that emerge early in development via the interplay of genetic and environmental factors. To better understand the neurobiology of temperament, we have selectively bred rats for generations to produce two lines with highly divergent behavior: bred Low Responders (bLRs) are highly inhibited and anxious in novel environments, whereas bred High Responders (bHRs) are highly exploratory, sensation-seeking, and prone to drug-seeking behavior. Recently, we delineated these heritable differences by intercrossing bHRs and bLRs (F_0_-F_1_-F_2_) to produce a heterogeneous F_2_ sample with well-characterized lineage and behavior (exploratory locomotion, anxiety-like behavior, Pavlovian conditioning). The identified genetic loci encompassed variants that could influence behavior via many mechanisms, including proximal effects on gene expression. Here we measured gene expression in male and female F_0_s (*n* = 12 bHRs, 12 bLRs) and in a large sample of heterogeneous F_2_s (*n* = 250) using hippocampal RNA-Seq. This enabled triangulation of behavior with both genetic and functional genomic data to implicate specific genes and biological pathways. Our results show that bHR/bLR differential gene expression is robust, surpassing sex differences in expression, and predicts expression associated with F_2_ behavior. In F_0_ and F_2_ samples, gene sets related to growth/proliferation are upregulated with bHR-like behavior, whereas gene sets related to mitochondrial function, oxidative stress, and microglial activation are upregulated with bLR-like behavior. Integrating our F_2_ RNA-Seq data with previously-collected whole genome sequencing data identified genes with hippocampal expression correlated with proximal genetic variation (*cis*-expression quantitative trait loci or *cis*-eQTLs). These *cis*-eQTLs successfully predict bHR/bLR differential gene expression based on F_0_ genotype. Sixteen of these genes are associated with *cis*-eQTLs colocalized within loci we previously linked to behavior and are strong candidates for mediating the influence of genetic variation on behavioral temperament. Eight of these genes are related to bioenergetics. Convergence between our study and others targeting similar behavioral traits revealed five more genes consistently related to temperament. Overall, our results implicate hippocampal bioenergetic regulation of oxidative stress, microglial activation, and growth-related processes in shaping behavioral temperament, thereby modulating vulnerability to psychiatric and addictive disorders.

## 1 Introduction

Psychiatric disorders can be classified using an internalizing versus externalizing model (Cerdá et al., 2010; Kendler et al., 1992; Krueger and Markon, 2006). Internalizing disorders are characterized by negative emotion, including depression, anxiety, and phobias, whereas externalizing disorders are characterized by behavioral disinhibition, including conduct disorder, antisocial behavior, and impulsivity. These internalizing and externalizing tendencies are associated with personality or temperament traits, such as neuroticism and sensation-seeking, that emerge early in development and are highly heritable (Bienvenu et al., 2001; Caspi et al., 1996; Clark et al., 1994; Jardine et al., 1984; Kagan and Snidman, 1999; Karlsson Linnér et al., 2021; Khan et al., 2005; Sanchez-Roige et al., 2018, 2019; Zuckerman and Cloninger, 1996; Zuckerman and Kuhlman, 2000). Thus, elucidating the genetic contribution to temperament could provide insight into the etiology of a variety of psychiatric and addictive disorders.

One compelling way to explore the genetic contribution to temperament is to selectively breed animals that show extreme behavioral traits. Selectively breeding laboratory rodents has confirmed the heritability of extreme anxiety-like and depressive-like behavior, risk-seeking, exploratory behavior, substance use, and hyperactivity (Almeida et al., 2018; Brush, 2003; Castanon et al., 1995; Commissaris et al., 1986; Filiou et al., 2014; Hendley et al., 1983; Jónás et al., 2010; Kessler et al., 2007; Overstreet et al., 1994; Rezvani et al., 2002; Terenina-Rigaldie et al., 2003; Weiss et al., 1998; Wigger et al., 2001). Within our laboratory, we have selectively bred rats for two decades for either a high propensity to explore a novel environment (high responders to novelty) or a low propensity to explore a novel environment (low responders to novelty) (Stead et al., 2006; Turner et al., 2017). We have found that this locomotor response to a novel environment (LocoScore) predicts a broader behavioral phenotype in our bred lines, akin to human temperament (Flagel et al., 2014; Turner et al., 2017). The bred high responders (bHRs) have high exploratory locomotion and disinhibited, sensation-seeking, externalizing-like behavior. They show greater impulsivity, low anxiety, and an active coping style. They are highly sensitive to reward cues, which can become attractive and reinforcing in a Pavlovian conditioned approach (PavCA) task (“sign-tracking”) (Flagel et al., 2011). In contrast, bred low responders (bLRs) have low exploratory locomotion and inhibited, internalizing-like behavior. They show elevated anxiety- and depressive-like behavior, stress reactivity, a passive coping style (Aydin et al., 2015; Clinton et al., 2014; Flagel et al., 2014, 2016; Turner et al., 2017), and primarily use reward cues for their predictive value (PavCA “goal-tracking”) (Flagel et al., 2011). These behavioral phenotypes appear early in development (Clinton et al., 2011; Turner et al., 2019) similar to temperament in humans (Mervielde et al., 2005; Saudino, 2005). Thus, the highly divergent bHR/bLR phenotypes model the heritable extremes in temperament predictive of internalizing and externalizing psychiatric disorders in humans. They can also model two paths to substance use disorders and addiction: sensitivity to reward cues and sensation-seeking makes bHRs more likely to initiate and re-initiate substance use, whereas bLRs increase substance use following stress (Flagel et al., 2014, 2016).

**Figure 1:**
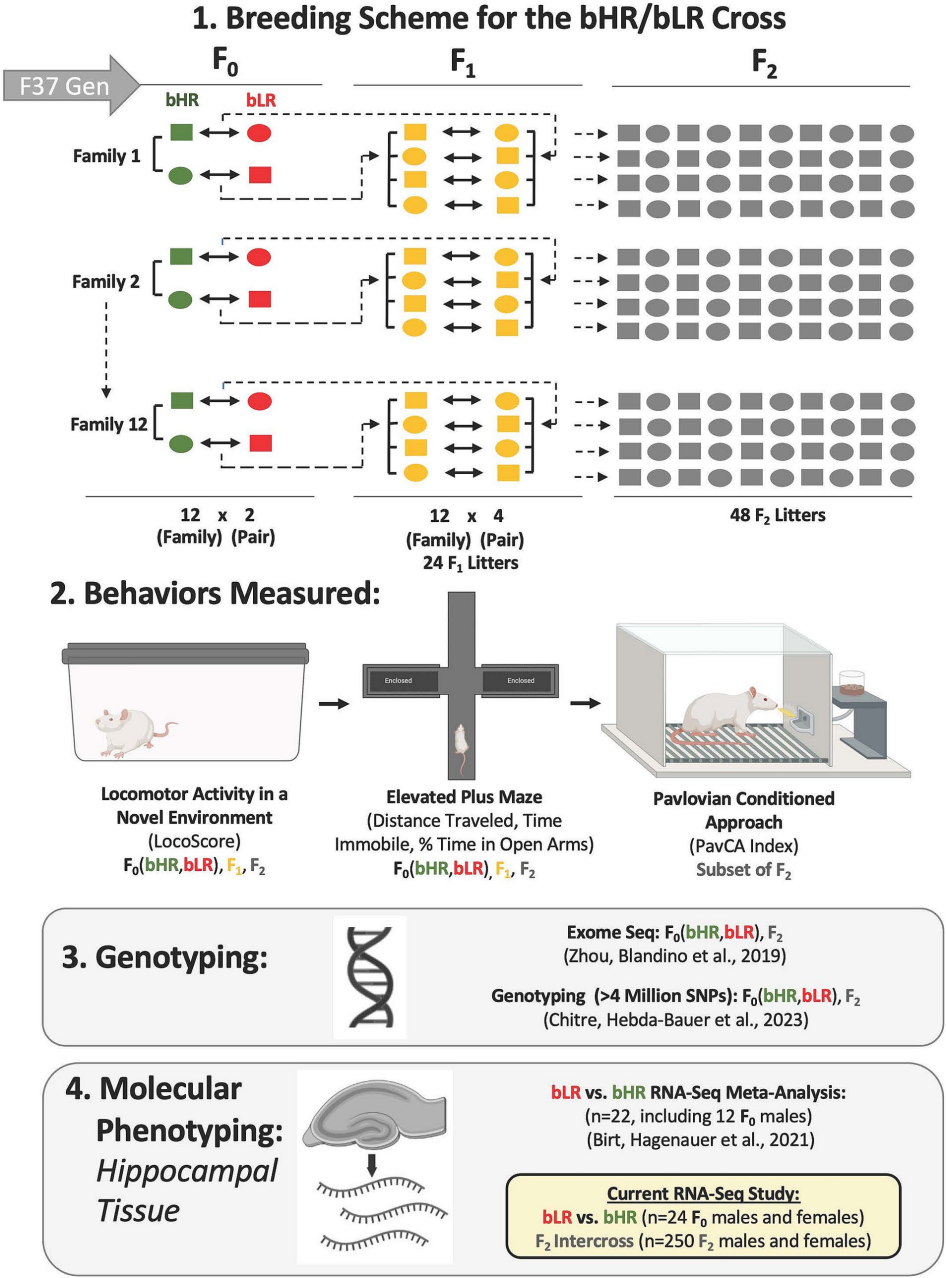
Experimental design: Crossbreeding bHR and bLR rats to identify genes implicated in exploratory, anxiety-like, and reward-related behaviors. After 37 generations of selectively breeding rats for a high or low propensity to explore a novel environment, we have generated two lines of rats [high responders to novelty (bHRs) or low responders to novelty (bLRs)] with highly divergent exploratory locomotion, anxiety-like behavior, and reward-related behavior [Pavlovian conditioned approach (PavCA)]. 1. Breeding Scheme: An initial set (F_0_) of bHRs were bred with bLRs to create 12 intercross families. The offspring of this intercross (F_1_) were then bred with each other to produce a re-emergence of diverse phenotypes in the F_2_ generation. 2. Behavior: All rats were assessed for locomotor activity in a novel environment (LocoScore) as well as exploratory and anxiety-like behavior in the elevated plus maze (EPM). For a subset of F_2_ rats, sensitivity to reward-related cues (Pavlovian Conditioned Approach (PavCA) behavior) was also measured. 3. Genotyping: To identify genomic loci associated with bHR/bLR phenotype (F_0_ population segregation) and behavior in F_2_ adults and F_2_ juveniles (quantitative trait loci, QTLs), exome sequencing was initially performed on both F_0_ and F_2_ rats (Zhou et al., 2019), followed by a broader genome wide association study (GWAS) (Chitre et al., 2023). Within the GWAS, the whole genome was deeply sequenced for the F_0_ rats. This whole genome sequencing (WGS) data was then used in conjunction with low pass (~0.25x) WGS data from the larger cohort of F_2_ rats to impute the genotype of 4,425,349 single nucleotide variants (SNPs) for each rat (Chitre et al., 2023). 4. Molecular Phenotyping: RNA-Seq was used to characterize gene expression in the hippocampus of a subset of male F_0_ and F_1_ rats (*n* = 6/subgroup), which was included in a cross-generational bHR/bLR meta-analysis (Birt et al., 2021). In our current study, RNA-Seq was used to characterize hippocampal gene expression in an independent set of males and females in the F_0_ (*n* = 24, *n* = 6 per phenotype per sex) and F_2_ (*n* = 250) rats to identify gene expression related to both bHR/bLR lineage and exploratory locomotion, anxiety-like behavior, and PavCA behavior.

The extreme divergence in bHR/bLR behavior makes them a powerful model for investigating the heritable contributions to temperament. However, like all selective breeding models, the bHR/bLR lines are likely to be enriched with genetic alleles contributing to the behavioral phenotype as well as alleles that are merely in linkage disequilibrium with the causal locus. To hone in on causal loci for our selected behavioral phenotype, we used a classic method of producing a series of crosses (F_0_-F_1_-F_2_) to generate a heterogeneous sample with well-characterized lineage. We bred bHRs with bLRs from generation 37 (F_0_) to produce F_1_ cross offspring (Intermediate Responders, “IR”). These F_1_ offspring were then bred with each other to produce a re-emergence of diverse behavioral phenotypes in the F_2_ generation (Figure 1). We then performed exome and whole genome sequencing on the F_0_ and F_2_ rats (Chitre et al., 2023; Zhou et al., 2019) to reveal coding differences segregating the bHR/bLR lines (F_0_s) and chromosomal regions associated with variation in exploratory and anxiety-related behaviors in the F_2_ adults and juveniles [quantitative trait loci (QTLs)]. However, each of the loci associated with behavior (QTLs) in the F_2_ rats still encompassed many genetic variants segregated in the bHR/bLR rats, potentially influencing the expression of multiple, diverse genes. Thus, additional studies were necessary to pinpoint gene expression that might mediate functional effects on the brain leading to bHR/bLR behavior. This step is important, because the implicated genetic variants themselves are not necessarily translatable across species, or even across strains, but can guide us to causal pathways.

Therefore, our goal in the current study was to obtain brain gene expression data from the F_0_ and F_2_ animals which could provide insight into the functional mechanisms mediating the influence of genetic variation on behavioral phenotype. We chose to focus on the hippocampus due to its importance in behaviors that diverge between our bred lines, including novelty processing, exploration, behavioral inhibition, emotional regulation, environmental reactivity, and stress-related responses (Campbell and Macqueen, 2004; Fanselow and Dong, 2010; Gerlach and McEwen, 1972; Gray, 1982; Johnson et al., 2012; Papez, 1937; Schwarting and Busse, 2017) The hippocampus has also been linked to the heritable component of anxious or inhibited temperament (Oler et al., 2010), and both internalizing and externalizing disorders (Campbell and Macqueen, 2004; Hoogman et al., 2017; Schmaal et al., 2016). Importantly, previous investigations found pronounced bHR/bLR differences in hippocampal function both in adulthood and early in development (Birt et al., 2021; Clinton et al., 2011; McCoy et al., 2019; Perez et al., 2009; Simmons et al., 2012; Turner et al., 2008; Widman et al., 2019), suggesting that it might be a key region in the generation of the phenotype.

**Figure 2:**
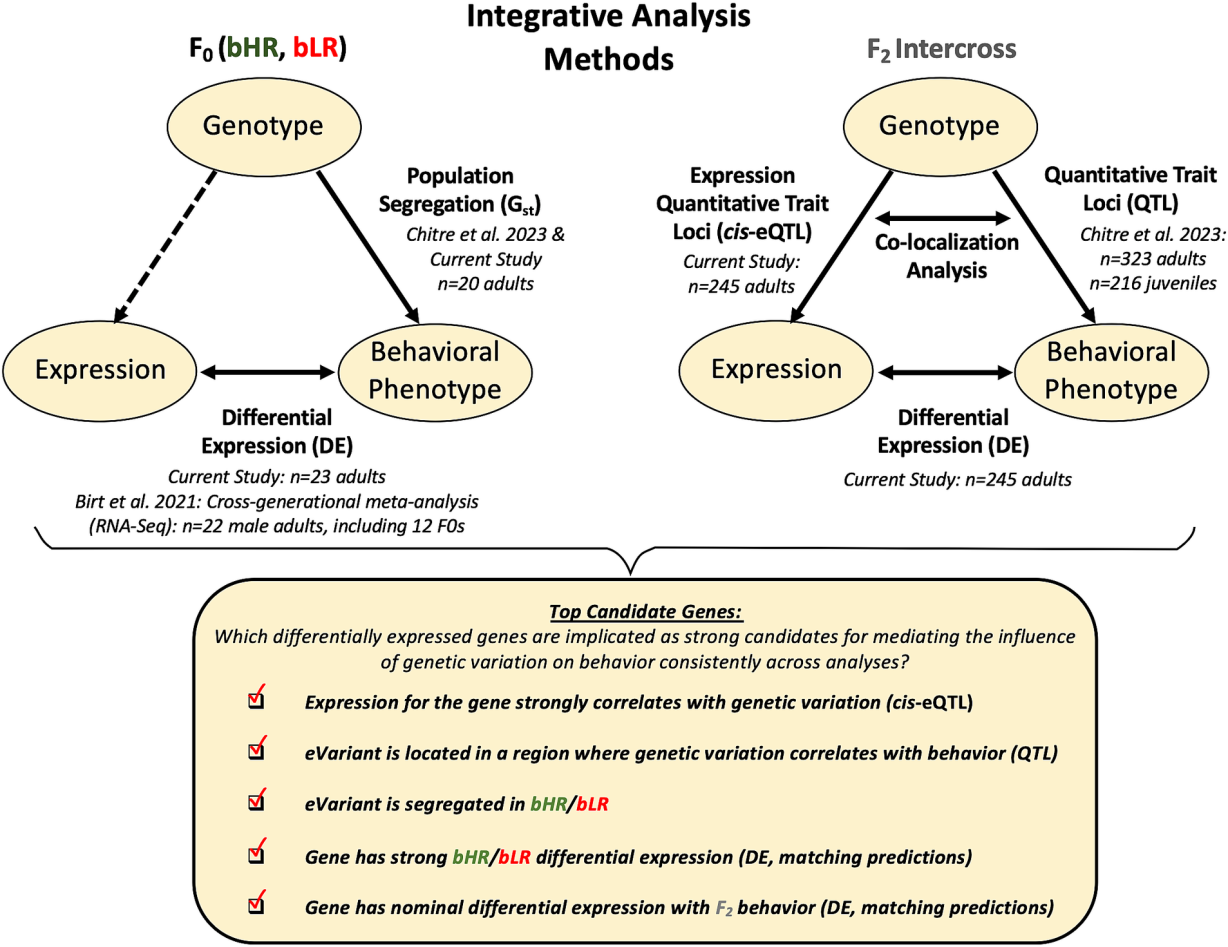
Analysis methods: Using convergent evidence to identify differentially expressed genes that are the strongest candidates for mediating the influence of genetic variation on behavioral temperament. We first identified expression in the hippocampus that differentiated the bHR and bLR lines, using information from both our current F_0_ sample and previous cross-generational meta-analysis. We then identified gene expression that continued to correlate with bHR/bLR divergent behaviors in our large F_2_ intercross sample, indicating that the differential expression (DE) was not an artifact of genetic drift. To determine which DE was most likely driven by proximal genetic variation, we performed a cis-expression Quantitative Trait Loci (cis-eQTL) analysis to determine which genes (eGenes) had expression that strongly correlated with nearby genetic variants (eVariants) using the F_2_ genotype and F_2_ gene expression data, and then estimated the magnitude of that effect in Log2 fold change units (allelic fold change or aFC). We determined which eVariants were segregated in the bHR/bLR lines and confirmed that the predicted effect of this genetic segregation on gene expression matched the bHR/bLR DE observed in the F_0_s. We also determined which eVariants co-localized in regions of the genome previously identified as having strong relationships with behavior in the full sample of F_2_ adults and F_2_ juveniles (QTLs) using Summary-Data Based Mendelian Randomization (SMR) and compared the predicted direction of effect of this genetic variation on gene expression to the DE observed in the F_2_ adults in association with behavior. Finally, to be considered a top candidate gene for mediating the influence of genetic variation on behavioral temperament, we required that a gene have hippocampal DE related to both bHR/bLR phenotype and F_2_ behavior which appeared plausibly driven by genetic variation (cis-eQTL) that segregated in the bHR/bLR lines and correlated with behavior (SMR colocalization with QTL). Note that the sample sizes listed in the diagram reflect the sample sizes used in the analysis following quality control.

To identify the genes and biological pathways that shape temperament, the present study triangulated the newly-collected functional genomics data with previously-collected behavioral and genetic data (Figure 2). We first used RNA-Sequencing of hippocampal tissue from both male and female bHRs and bLRs (F_0_, *n* = 24) to confirm and expand upon our earlier results from a cross-generational meta-analysis of hippocampal gene expression in bHR versus bLR males (Birt et al., 2021). We then performed RNA-Sequencing of hippocampal tissue from a large sample of heterogeneous F_2_ intercross rats (*n* = 250) to identify differential expression that continued to correlate with exploratory locomotion, anxiety-like behavior, and reward-related behavior independent of the linkage disequilibrium and genetic drift specific to our bred lines. To determine generalizability, we compared these results to hippocampal differential expression from other rat models and to bHR/bLR differential expression in other brain regions. Then, to determine which differential expression was most likely to be driven directly by genetic variation, we integrated our current F_2_ RNA-Seq data with previous whole genome sequencing results (Chitre et al., 2023) to identify genes with expression tightly correlated with proximal genetic variation (expression QTLs: *cis*-eQTLs). We determined which of these *cis*-eQTLs were segregated in the bHR/bLR lines and co-localized with the loci that we had previously linked to behavior (QTLs) within the larger F_2_ sample [adults and juveniles: Chitre et al., 2023]. This converging evidence revealed a set of differentially expressed genes that are particularly strong candidates for mediating the neurobiology of temperament.

## 2 Materials and methods

Full methods are in the Supplementary material, including the ARRIVE reporting checklist. Analysis code (R v.3.4.1-v.4.2.2, R-studio v.1.0.153-v.2022.12.0+353) has been released at https://github.com/hagenaue/NIDA_bLRvsbHR_F2Cross_HC_RNASeq.

All procedures were conducted in accordance with the National Institutes of Health Guide for the Care and Use of Animals and approved by the Institutional Animal Care and Use Committee at the University of Michigan.

### 2.1 Animals

Selectively breeding rats for high or low locomotor activity in a novel environment (LocoScore) produced the bHR line (Wakil:bHR, RRID:RGD_405847397) and bLR line (Wakil:bLR, RRID:RGD_405847400), respectively (Stead et al., 2006). After 37 generations, 12 bHRs and 12 bLRs (F_0_) were chosen from 24 distinct families to crossbreed. The F_1_ offspring with similarly high or low LocoScores were then bred with each other to produce a re-emergence of diverse behavioral phenotypes in the F_2_ generation (Figure 1). These 48 F_2_ litters generated 540 rats [*n* = 216 behaviorally phenotyped as juveniles (1 month old), *n* = 323 behaviorally phenotyped as young adults (2–4 months old)]. Our current study sampled a subset of the adults (F_0_: *n* = 24, *n* = 6/phenotype per sex; F_2_: *n* = 250, *n* = 125/sex) that overlapped with previous genetic studies (Chitre et al., 2023; Zhou et al., 2019) but was distinct from our previous male-only meta-analysis (Birt et al., 2021).

### 2.2 Behavioral analysis

Behavioral phenotyping for the F_0_ and F_2_ rats used in our current study was performed in adulthood, in the morning, with each test occurring on separate days. Testing order was counterbalanced for phenotype (bHR/bLR), with males tested before females on separate days. All F_0_ and F_2_ rats were tested for locomotor activity in a novel environment (protocol: Stead et al., 2006). During testing, rats were placed in a box akin to their home cage but located in a different room with novel cues. Over 60 min, lateral and rearing movements were counted via beam breaks, and the cumulative total defined as locomotor score (LocoScore). All F_0_ and F_2_ were tested for exploratory and anxiety-related behaviors on an EPM [dimly lit (40 lux), protocol: Chitre et al., 2023]. Rats began the five-minute test at the intersection of the arms. A video tracking system (Ethovision, Noldus Information Technology) recorded the percent time spent in the open and closed arms, distance traveled (cm), and time immobile (sec). A subset of F_2_s (*n* = 209) subsequently underwent seven sessions of PavCA training to measure their bias in favor of reward cues over the reward itself (protocol: Meyer et al., 2012). To create a summary PavCA Index, three behavioral variables were averaged: (1) *Probability difference*: the probability of lever contact minus the probability of food magazine entries, (2) *Response bias*: the total conditioned stimuli (CS: lever) contacts minus the total food magazine entries, divided by the sum of the two behaviors. (3) *Latency score*: the average latency to enter the food magazine minus the average latency to contact the lever, divided by the length of the CS duration (8 s). The PavCA Index for the last 2 days of testing (days six and seven) was used to classify rats as “sign trackers” (ST: values > 0.5), “intermediate” (IN: values −0.5 to 0.5), or “goal trackers” (GT: values < −0.5).

For each of the continuous behavioral variables, the interacting effects of sex and phenotype (F_0_ bHR vs. F_0_ bLR vs. F_2_) were examined using analysis of variance (ANOVA type 3, contrasts = “contr.sum”). Exploratory analyses were also performed to examine the potential effect of batch-related variables, testing order, maternal lineage, and paternal lineage. Correlations between behaviors were characterized in the F_0_ and F_2_ datasets separately using parametric methods (Pearson’s R, simple linear model). For the F_0_s, Lineage was included as a dummy variable (bHR as reference 0, bLR coded as 1). For PavCA, a Fisher’s Exact Test was performed on the ratios of male to female animals classified as ST, IN, or GT.

### 2.3 Tissue dissection, RNA extraction, and sequencing

Adults (postnatal days 113–132) were decapitated without anesthesia and brains rapidly extracted (<2 min). For the F_0_s, whole hippocampus was immediately dissected and flash frozen. For the F_2_s, whole brains were flash frozen, and later hole punches from the dorsal and ventral hippocampus were pooled from four hemisected coronal slabs per rat (−2.12 to −6.04 mm Bregma; Paxinos and Watson, 2013). RNA was extracted using the Qiagen RNeasy Plus Mini Kit. A NEB PolyA RNA-seq library was produced and sequenced using a NovaSeq S4 101PE flowcell (targeting 25 million reads/sample).

### 2.4 Hippocampal RNA-Seq analysis

RNA-Seq data preprocessing was performed using a standard pipeline including alignment (STAR 2.7.3a: genome assembly Rnor6), quantification of gene level counts (Ensembl v103, Subread version 2.0.0), and basic quality control. All downstream analyses were performed in Rstudio (v.1.4.1717, R v. 4.1.1). Transcripts with low-level expression (<1 read in 75% of subjects) were removed. Normalization included the trimmed mean of M-values (TMM) method (Robinson and Oshlack, 2010, *edgeR* v.3.34.1; Robinson et al., 2010), and transformation to Log2 counts per million [Log2 cpm (Law et al., 2016), *org.Rn.eg.db* annotation v.3.13.0; Carlson, 2019]. Following quality control, the F_0_ dataset contained *n* = 23 subjects (subgroups: *n* = 5 bHR females, *n* = 6 for each of the other subgroups: bHR males, bLR females, bLR males) with Log2 cpm data for 13,786 transcripts, and the F_2_ dataset contained *n* = 245 subjects (subgroups: *n* = 122 males, *n* = 123 females) with Log2 cpm data for 14,056 transcripts.

Differential expression was calculated using the limma/voom method (Law et al., 2014, package: *limma* v.3.48.3) with empirical Bayes moderation of standard error and FDR correction. For the F_2_s, the same differential expression model was used for each variable of interest (LocoScore, EPM % time in open arms, EPM distance traveled, EPM time immobile, PavCA Index). Technical co-variates were included if they were strongly related to the top principal components of variation identified using Principal Components Analysis (PCA) or had confounding collinearity with variables of interest [covariates: percent of reads that were intergenic (%intergenic) or ribosomal RNA (%rRNA), dissector, sequencing batch, and PavCA exposure (“STGT_Experience”)].

Equation 1: F_0_ differential expression model:


$$y\sim \beta_{0}+\beta_{1}\mathrm{Lineage}+\beta_{2}\mathrm{Sex}+\beta_{3}\%\mathrm{rRNA}+\beta_{4}\%\mathrm{Intergenic}+\varepsilon$$ (1)

Equation 2: F_2_ differential expression model:


$$\begin{array}{l} y\sim \beta_{0}+\beta_{1}\mathrm{VariableOfInterest}+\beta_{2}\mathrm{Sex}+\beta_{3}\%\mathrm{rRNA}+\\ \beta_{4}\%\mathrm{Intergenic}+\beta_{5}\mathrm{Dissector}+\beta_{6}\mathrm{STGT}\_\\ \mathrm{Experience}+\beta_{7-8}\mathrm{SequencingBatch}+\varepsilon \end{array}$$ (2)

### 2.5 Comparison of F_0_, F_2_, and previous hippocampal meta-analysis results

The full F_0_ and F_2_ differential expression results were compared to our previous meta-analysis of hippocampal RNA-Seq studies from late generation bHR/bLR males (Supplementary Table S2 in Birt et al., 2021) using parametric and non-parametric methods (Pearson’s and Spearman’s correlation of Log2FC values) and visualized using gene rank-rank hypergeometric overlap [*RRHO v. 1.38.0* (Plaisier et al., 2010; Rosenblatt and Stein, 2014), ranking by t-statistics] and *VennDiagram* [v.1.7.3 (Chen, 2022)]. For downstream analyses, we defined bHR/bLR differentially expressed genes as the 1,063 genes with FDR < 0.10 in either the F_0_s or late generation meta-analysis, or nominal replication (*p* < 0.05) in both with consistent direction of effect.

### 2.6 Comparison of bHR/bLR hippocampal results to findings from other regions

As an exploratory analysis, we compared bHR/bLR hippocampal differential expression to the pattern of differential expression in other brain regions in previous small transcriptional profiling studies of bHR/bLR adults, including publicly available data from the amygdala [GSE88874: *n* = 5/group, generation F31 (Cohen et al., 2015; McCoy et al., 2017), GSE86893: *n* = 6/group, generation F34-F36 (Cohen et al., 2017)] and dorsal raphe [GSE86893, *n* = 6/group (Cohen et al., 2017)], and unpublished data from the cortex (GSE286181, *n* = 6/group) and hypothalamus (GSE286181, *n* = 6/group) from an early generation of selective breeding (F4). To run this comparison, differential expression was calculated for each dataset using the *limma* pipeline (Supplementary methods). To reduce noise and increase statistical power, we used a standardized pipeline (Hagenauer M. et al., 2024) to perform a simple random effects meta-analysis (Viechtbauer, 2010, package: *metafor*) to summarize the amygdala differential expression results (collective sample size of *n* = 11/group for 7,133 genes), and—to potentially identify bHR/bLR differences that might exist brain-wide—all non-hippocampal differential expression results (collective *n* = 29/group for 11,509 genes). These results were compared to our hippocampal findings using non-parametric methods [Spearman’s correlation of Log2FC values and *RRHO v. 1.38.0* (Plaisier et al., 2010; Rosenblatt and Stein, 2014), ranking by t-statistics].

### 2.7 Gene set enrichment analysis

To elucidate functional patterns, we ran Gene Set Enrichment Analysis (*fgsea v.1.2.1*, nperm = 10,000, minSize = 10, maxSize = 1,000, FDR < 0.05) using a custom gene set database (Brain.GMT v.1, Hagenauer M. H. et al., 2024) that included standard gene ontology, brain cell types, regional signatures, and differential expression results from public databases. We created a continuous variable representing bLR-like vs. bHR-like differential expression for each gene by averaging the t-statistics for bLR vs. bHR comparisons in the F_0_ dataset and former late generation RNA-Seq meta-analysis (Birt et al., 2021) and for each of the F_2_ behaviors (with bHR-like phenotype set as reference). A second non-directional analysis used the absolute value of the average t-statistic.

### 2.8 Constructing a hippocampal *cis*-eQTL database

Hippocampal *cis-*eQTL mapping was performed using published methods Munro et al. (2022, unpublished)^1^. Quality-controlled F_2_ RNA-Seq data (Log2 CPM, *n* = 245 following quality control) was corrected for technical covariates (Equation 2, residualized), followed by rank-based inverse normal transformation ((see footnote 1) Ongen et al., 2016). F_2_ genotypes were generated by low coverage whole genome sequencing followed by imputation (from data release for (Chitre et al., 2023): doi: 10.6075/J0K074G9, *n* = 4,425,349 SNPs). Principal Components Analysis was run on the gene expression matrix and genotype matrix [following pruning for linkage disequilibrium, *Plink2 v.2.00a2.3* (Chang et al., 2015)], and principal components 1–5 from both analyses included as covariates within the single-SNP linear regression for *cis*-eQTL mapping [*tensorQTL v.1.0.6* (Taylor-Weiner et al., 2019)]. We tested SNPs with minor allele frequency (MAF) >0.01 within ±1 Mb of each gene’s transcription start site (tss). A significant eVariant-eGene relationship (*cis*-eQTL) was defined using empirical beta-approximated *p*-values calculated using permutations for each gene, and false discovery corrected (FDR < 0.05) using results from the top SNP for all genes. When SNPs were in perfect linkage disequilibrium, a single SNP was selected randomly. Additional, conditionally independent *cis*-eQTLs for each eGene were identified using stepwise regression (*tensorQTL:* default settings). We estimated *cis*-eQTL effect size (allelic fold change, aFC) using an additive *cis*-regulatory model (package *aFC.py*) with raw expression read counts and the same covariates as *cis*-eQTL mapping (Mohammadi et al., 2017).

### 2.9 Predicting bHR/bLR differential expression using the *cis*-eQTL database

We extracted F_0_ genotype information for each eVariant (*n* = 10 bHR/*n* = 10 bLR in Chitre et al., 2023, release: doi: 10.6075/J0K074G9) using *VcfR* (v1.14.0, Knaus and Grünwald, 2017).^2^ We defined partial bHR/bLR segregation using *myDiff()* G_st’_ > 0.27 [(Hedrick, 2005), akin to all 0/0 vs. all 0/1 in our dataset]. We assigned the direction of effect for the Log2 aFC to reflect the bLR-enriched allele vs. bHR-enriched allele and compared these predictions to both the F_0_ differential expression results (Log2FC) and bHR/bLR late generation RNA-Seq meta-analysis results (estimated *d*) using parametric (linear regression) and non-parametric (Spearman’s rho) methods.

### 2.10 Co-localization of *cis*-eQTLs with regions of the genome associated with bHR/bLR-like behavior

We used Summary Data-based Mendelian Randomization (SMR; Zhu et al., 2016) to test for colocalization of *cis*-eQTLs with QTLs from the full F_2_ cohort [GWAS results: (Chitre et al., 2023)] for adult behaviors included in our differential expression analysis (LocoScore, EPM time immobile, EPM distance traveled, EPM % time in open arms, PavCA Index) and juvenile behaviors targeting analogous traits (open field (OF) time immobile, OF distance traveled, OF % time in center). *Z*-scores for the *cis*-eQTL and GWAS associations with each top eVariant were used to calculate the SMR approximate chi-squared test statistic, with *p*-values determined using the chi-squared distribution’s upper tail [df = 1, FDR correction: *mt.rawp2adjp()* (proc = “BH”) in *multtest* v.2.26.0 (Pollard et al., 2005)]. Results were visualized using the *manhattan()* plot function in the *qqman* package [v.0.1.8 (Turner, 2018)].

To determine whether the strength of *cis*-eQTL/QTL co-localization (SMR t-statistic) correlated with F_2_ behavioral differential expression, we assigned a predicted direction of effect based on the relationship between genotype and behavior within the larger F_2_ sample (adults: *n* = 323 adults, juveniles: *n* = 216) and genotype and expression within the *cis*-eQTL analysis (*n* = 245). We then examined the correlation between the F_2_ Log2FCs for each adult behavior and the “directional” SMR T-statistics for the same adult behavior or analogous juvenile behavior (OF distance traveled, OF time immobile, OF % time in center), both in the full dataset (all 5,937 cis-eQTLs) and within the subset of *cis*-eQTLs that we had already confirmed were segregated in bHR/bLRs in a manner predictive of bHR/bLR differential expression (492 cis-eQTLs representing 456 eGenes).

To narrow down our pool of top candidate genes for mediating the effect of genetic variation on behavior, we required that our final top candidate genes have expression strongly related to genetic variation (*cis*-eQTLs) that is segregated in bHR/bLR (G_st’_ > 0.27) in a manner that correctly predicts bHR/bLR differential expression and is co-localized with a QTL for behavior (SMR FDR < 0.10) in a manner that correctly predicts F_2_ differential expression. Using a conservative estimate (Supplementary methods), this convergence of results should only be observable once, at most, in our dataset due to random chance.

## 3 Results

### 3.1 Locomotor activity in a novel environment reflects a broader behavioral temperament in both selectively-bred bHR/bLR lines and F_2_ intercross rats

**Figure 3:**
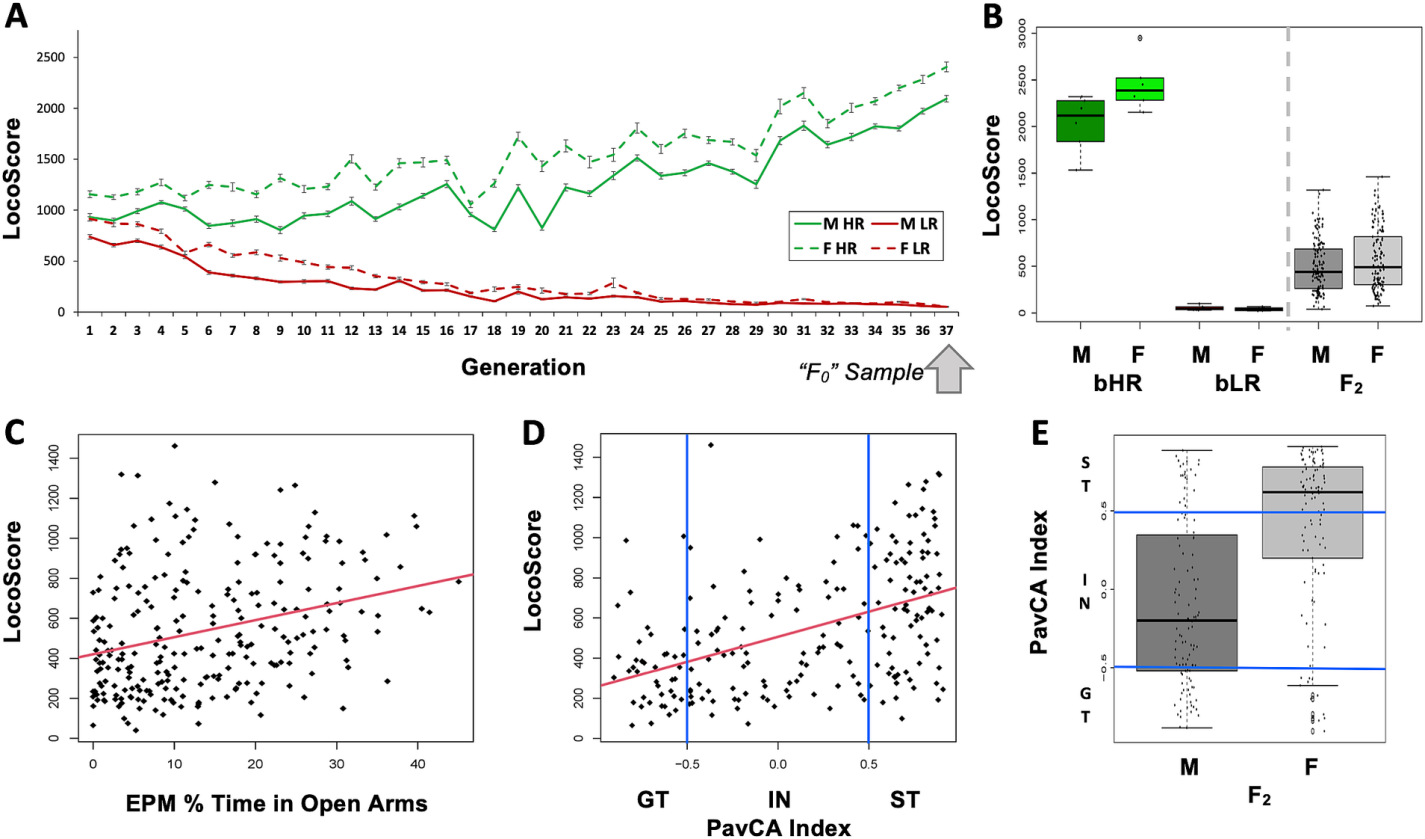
Behavioral phenotype: Locomotor activity in a novel environment reflects a broader behavioral temperament in both selectively-bred bHR/bLR lines and F_2_ intercross rats. **(A)** Locomotion in a novel environment (LocoScore: lateral + rearing counts over 60 min) is strongly influenced by genetics, as indicated by the divergence in LocoScore observed following the selective breeding of the bHR/bLR lines in both males (M) and females (F). The “F_0_” animals used in our crossbreeding experiment were taken from the 37^th^ generation of selective breeding. **(B)** The bHR/bLR (F_0_) crossbreeding scheme produced F_2_ rats with intermediate behavior which fell between the extreme bHR and bLR phenotypes. Depicted are the LocoScores for the F_0_ and F_2_ rats included in the current RNA-Seq experiment (effect of group: *F*(2, 268) = 212.764, *p* < 2.2E-16). The graphs for other measured behaviors (EPM Distance traveled, EPM time immobile, EPM % Time in Open Arms) are in Supplementary Figure S4. For all measured behaviors, group differences (ANOVA: F_0_ bHR vs. F_0_ bLR vs. F_2_) were highly significant (*p* < 1.5e-06, Supplementary Table S1). **(C,D)** Behaviors that diverged during bHR/bLR selective breeding for LocoScore remained correlated in F_2_s. Example scatterplots illustrate correlations between LocoScore and other bHR/bLR distinctive behaviors in the F_2_s. Red lines illustrate the relationship between the variables across both sexes. The correlations between all variables can be found in Supplementary Figure S4 and Supplementary Table S2. **(C)** Greater LocoScore predicted greater EPM % Time in Open Arms in the F_2_s (R = 0.30, *p* = 1.72E-06). Greater time spent in the open arms of the EPM is typically interpreted as indicating low anxiety. **(D)** Most F_2_s (*n* = 209) were tested for PavCA behavior. Greater LocoScore predicted an elevated PavCA Index in the F_2_s (R = 0.46, *p* = 3.22E-12). Rats with greater PavCA Index (>0.5) are considered sign-trackers (ST) and rats with lower PavCA Index (<−0.5) are considered goal-trackers (GT). **(E)** All behavioral variables showed a significant sex difference (*p* < 0.007) except for LocoScore. As an example, sex differences in PavCA index are illustrated with a boxplot. A higher percent of females than males were classified as Sign Trackers (ST) vs. Goal Trackers (GT) (Intermediate = IN) (Fisher’s exact test: *p* = 1.472E-06, OR: 0.13; CI: 0.05–0.34). These observed behavioral sex differences are difficult to interpret, as the two sexes were tested on all tasks in separate batches but supported the inclusion of sex as a covariate in all statistical models.

The bHR/bLR (F_0_) crossbreeding scheme produced F_2_ animals with behaviors ranging between the more extreme bHR and bLR phenotypes [all behaviors: *p* < 1.5e-06 for effect of group (F_0_ bHR vs. F_0_ bLR vs. F_2_); examples: Figures 3A,B; Supplementary Figure S4, full statistics: Supplementary Table S1]. F_2_ behavior sometimes appeared more similar to bLRs than bHRs (e.g., Figure 3B) suggesting a floor effect or that genetic contributions to internalizing-like behavior may be more dominant.

Exploratory locomotion, anxiety-like behavior, and PavCA behavior remained strongly correlated within the F_2_s, as previously observed in the bHR/bLR lines (examples: Figures 3C,D; Supplementary Figure S4, full statistics: Supplementary Table S2). Although these behaviors often differed by sex (example: Figure 3E, Supplementary Figure S4, full statistics: Supplementary Table S1), sex differences were not responsible for driving the correlation between different behaviors (with sex in the model: all behavior–behavior relationships still *p* < 0.0284). These findings are consistent with results using the full F_2_ cohort (Chitre et al., 2023) and imply that locomotor activity in a novel environment echoes a broader behavioral temperament, reflecting genetic and environmental influences shared across anxiety, mood, and reward-related behaviors.

### 3.2 F_0_ RNA-Seq: selective breeding produced a robust molecular phenotype in the hippocampus that surpassed the effect of sex

**Figure 4:**
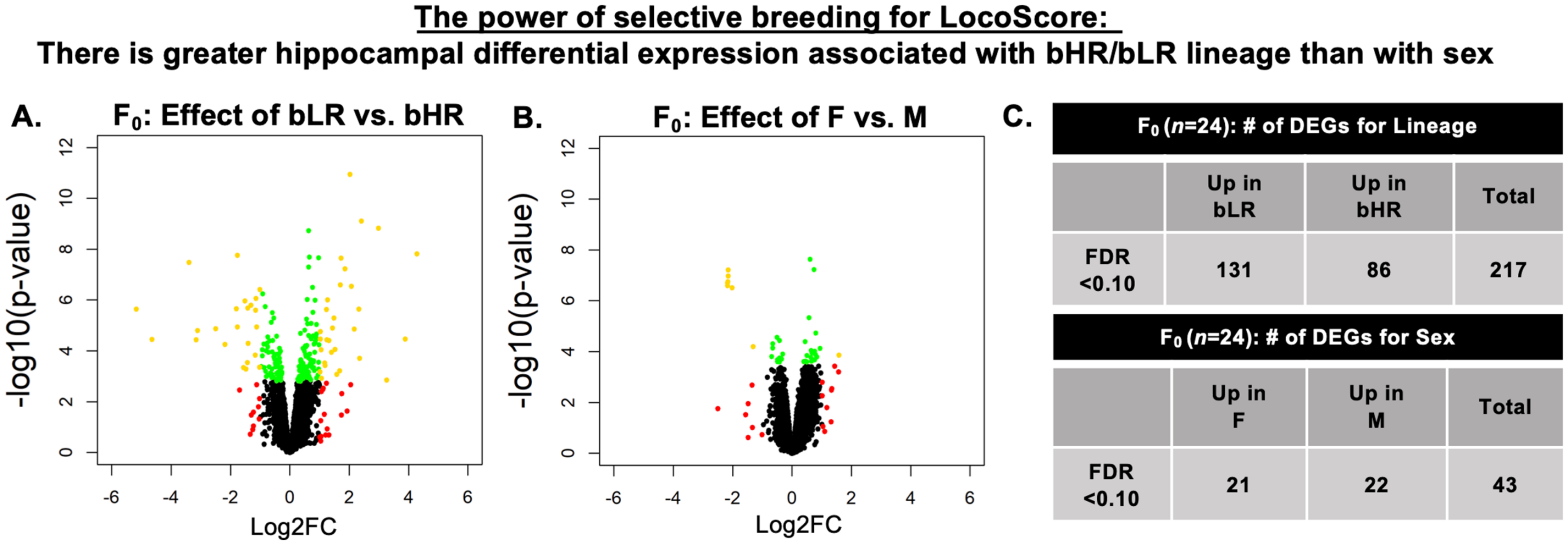
A robust hippocampal (HPC) molecular phenotype: There was greater hippocampal differential expression associated with F_0_ bHR/bLR lineage than with sex. Differential expression associated with bHR/bLR Lineage and Sex were examined in the same dataset (F_0_) with comparable subgroup sample sizes (total *n* = 23). Shown are two volcano plots illustrating the differential expression associated with bHR/bLR phenotype **(A)** and sex **(B)**. For both volcano plots, red depicts genes with a log2 fold change (Log2FC) > 1.0, green depicts genes with a False Discovery Rate (FDR) < 0.1, and gold indicates genes satisfying both criteria. In **(A)**, the reference group was defined as bHR, therefore positive Log2FC coefficients indicate upregulation in bLRs, and negative Log2FC coefficients indicate upregulation in bHRs. In **(B)**, males served as the reference group, therefore positive Log2FC coefficients indicate upregulation in females (F), and negative Log2FC coefficients indicate upregulation in males (M). For ease of visualization, six X and Y chromosome genes were not plotted due to extreme *p*-values (ranging from *p* = 5.71E-13 to 8.97E-26: Kdm5d, Eif2s3y, Uty, Ddx3, ENSRNOG00000055225, AABR07039356.2). The summary table **(C)** shows the number of differentially expressed genes (DEGs) for bHR/bLR Lineage and Sex. The full F_0_ bHR/bLR differential expression results can be found in Supplementary Table S3 and the full F_0_ differential expression results for Sex can be found in Supplementary Table S4.

The hippocampus plays an important role in many processes relevant to bHR/bLR behavioral phenotype, including novelty processing, exploratory behavior, behavioral inhibition, emotional regulation, environmental reactivity, and stress-related responses. We observed robust bHR/bLR differential expression in the hippocampus. Within the F_0_ RNA-Seq dataset, there were 131 differentially expressed genes with elevated expression in bLRs versus bHRs, and 86 differentially expressed genes with higher expression in bHRs (False Detection Rate (FDR) < 0.10, Figures 4A,C, Supplementary Table S3). In contrast, despite the observed sex differences in behavior, there were only 21 genes upregulated in females (versus males) and 22 genes upregulated in males (versus females) (Figures 4B,C, Supplementary Tables S3, S4). The effect sizes (Log(2) Fold Changes, or Log2FC) for bHR/bLR differentially expressed genes were also larger than those for sex, with the exception of a few X and Y chromosome genes (Figures 4A,B, Supplementary Tables S3, S4). There were no significant interactions between the effects of Lineage and Sex on gene expression (FDR > 0.10), but our sample size was underpowered to detect these effects (*n* = 5−6/subgroup). The presence of robust bHR/bLR hippocampal differential expression in both male and female F_0_s replicated previous male-only studies (Birt et al., 2021).

### 3.3 Current F_0_ study replicated bHR/bLR gene expression differences detected in previous studies

**Figure 5:**
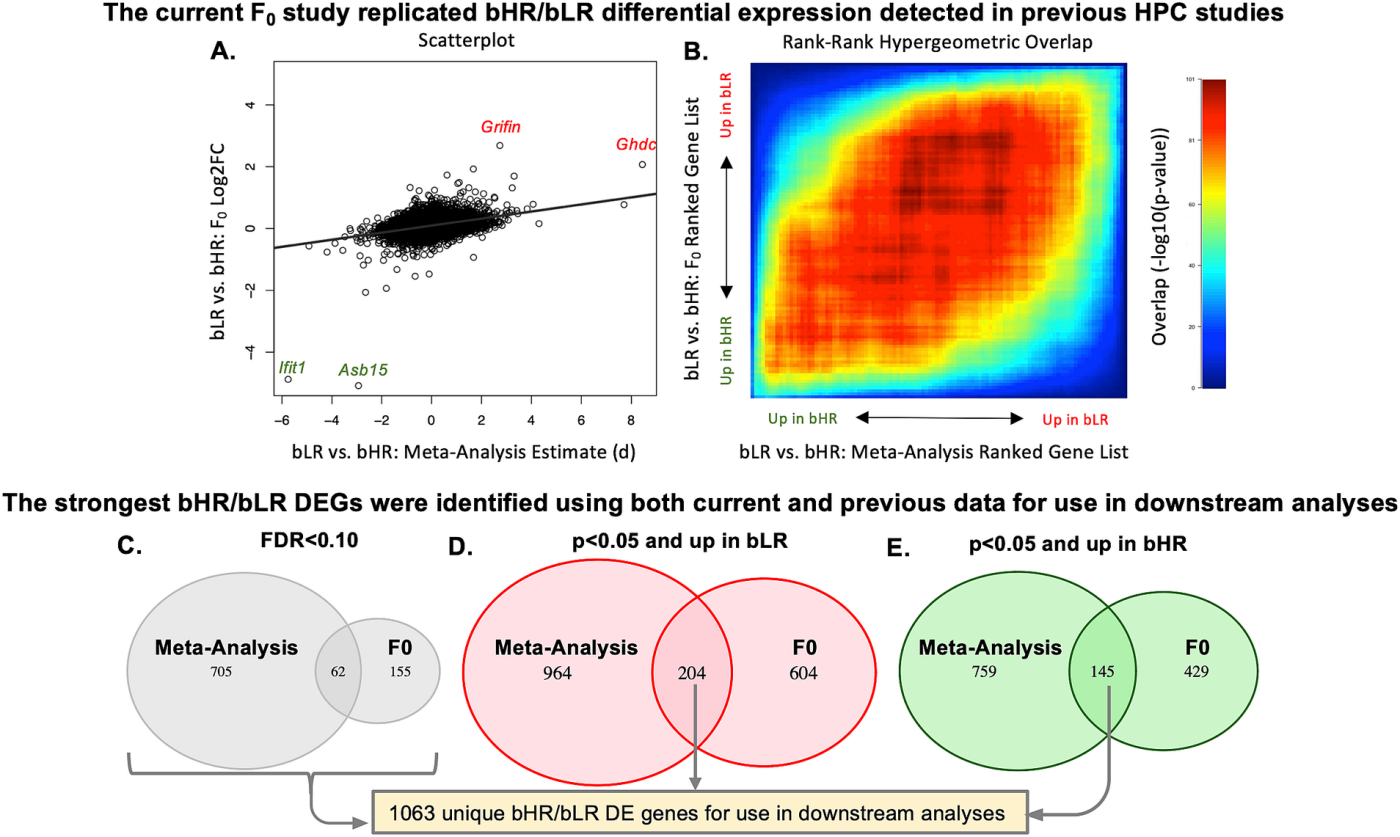
The current F_0_ study in males and females replicated bHR/bLR differential expression (DE) detected in previous male-only hippocampal (HPC) studies. **(A)** A scatterplot illustrates the positive correlation (*n* = 11,175 genes, R = 0.35, *p* < 2.2E-16) between the bHR/bLR effect sizes from our current F_0_ dataset (bLR vs. bHR Log2FC for all genes) and the bLR vs. bHR effect sizes identified by our previous late generation RNA-Seq meta-analysis ((Birt et al., 2021): bLR vs. bHR estimated Cohen’s d for all genes). Genes with particularly large effects in both datasets are labeled in green (upregulated in bHRs) or red (upregulated in bLRs). **(B)** This positive correlation is also visible when using a non-parametric analysis of the results ranked by t-statistic, as illustrated with a two-sided Rank-Rank Hypergeometric Overlap (RRHO) plot. Warmer colors illustrate the strength of the overlap [−log10(*p*-value)], with the visible red diagonal indicating a positive correlation between the ranked results. **(C–E)** To identify the strongest bHR/bLR differentially expressed genes (DEGs) for use in downstream analyses, we referenced results from both the current F_0_ dataset and previous late generation RNA-Seq meta-analysis (Birt et al., 2021). Shown are three Venn diagrams illustrating the overlap of the DEG lists from the two studies, with DE defined either using a traditional threshold of FDR < 0.10 in either study **(C)** or using a nominal *p*-value threshold (*p* < 0.05) and a specified direction of effect [**(D)**: upregulated in bLR vs. bHR, **(E)**: upregulated in bHR vs. bLR]. In each case, the overlap exceeded what would be expected due to random chance (OR > 4.7, *p* < 2.2e-16). The 1,063 unique genes with either FDR < 0.10 in either study or nominal replication with a consistent direction of effect in both studies were considered to have the strongest evidence of bLR vs. bHR DE and highlighted in downstream analyses.

The bHR/bLR hippocampal differential expression in our current study replicated many effects observed in our previous meta-analysis of hippocampal transcriptional profiling studies in bHR/bLR males (Birt et al., 2021), with the F_0_ Log2FC correlating positively with the bLR versus bHR estimated effect size (d) observed in RNA-Seq data from later generations (Figures 5A,B). Sixty-two of the 984 bHR/bLR differentially expressed genes in either dataset (FDR < 0.10) were significant (FDR < 0.10) in both datasets (Figure 5C, Supplementary Table S3). More genes showed replication of nominal bHR/bLR effects (*p* < 0.05) with consistent direction of effect in both datasets, so that, in total, 1,063 genes had evidence of bHR/bLR differential expression in the hippocampus (Figures 5D,E, Supplementary Table S3). As many generations of selective breeding for a behavioral phenotype are likely to produce an enrichment of eQTL alleles influencing the phenotype, these 1,063 genes were prioritized in downstream analyses.

As an exploratory analysis, we also compared the pattern of bHR/bLR differential expression identified in the hippocampus in our current study to bHR/bLR differential expression in other brain regions using data from previous transcriptional profiling studies in bHR/bLR adults, including the amygdala [*n* = 5/group (Cohen et al., 2015; McCoy et al., 2017), *n* = 6/group (Cohen et al., 2017)], dorsal raphe [*n* = 6/group (Cohen et al., 2017)], and unpublished data from the cortex (*n* = 6/group, Supplementary Table S5) and hypothalamus (*n* = 6/group, Supplementary Table S5). To increase power, we performed a meta-analysis of the two amygdala datasets (collective *n* = 11/group, 7,133 genes, Supplementary Table S5) and a meta-analysis encompassing all of the non-hippocampal data to identify bHR/bLR differences that might exist brain-wide (*n* = 29/group, 11,503 genes, Supplementary Table S5). These comparisons suggested that at least some of the bHR/bLR differential expression identified in the hippocampus may also be present in other brain regions, whereas other differential expression may be hippocampal specific (Supplementary results).

### 3.4 F_0_ hippocampal differential expression predicts expression related to F_2_ behavior

**Figure 6:**
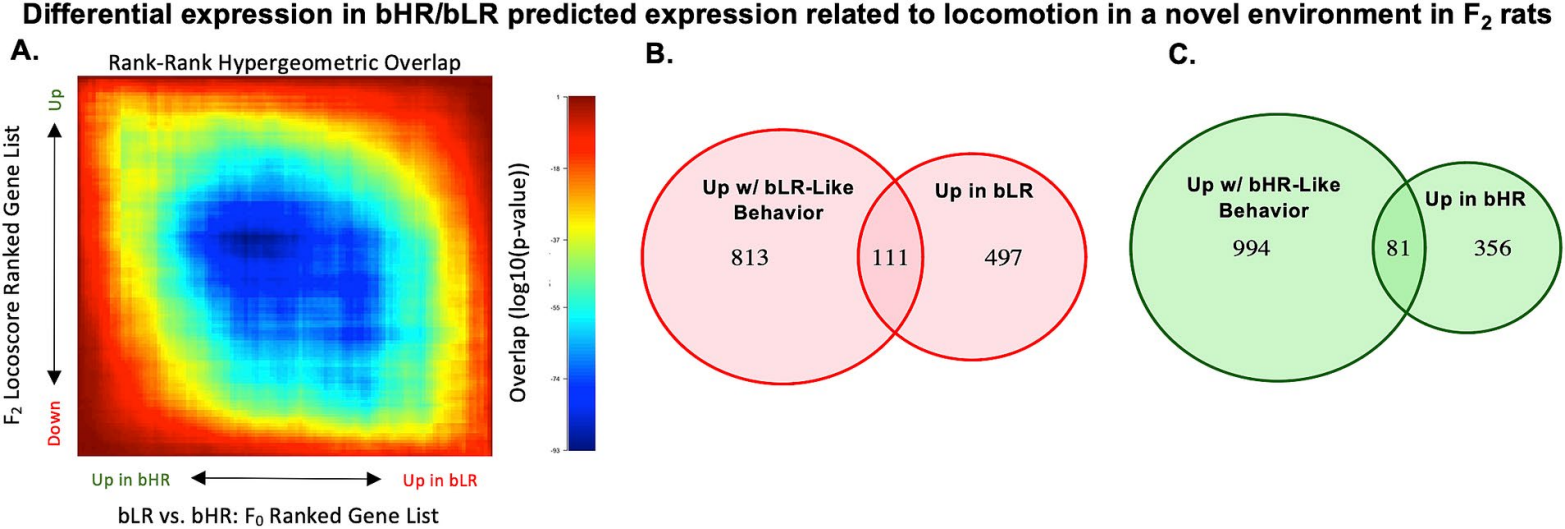
Hippocampal gene expression in bLR vs. bHR F_0_ rats predicts the pattern of gene expression associated with bLR-like vs. bHR-like behavior in F_2_ intercross rats. **(A)** For example, there is a negative correlation between the Log2FC associated with locomotion in a novel environment (LocoScore) in the F_2_s and the Log2FC for bLR vs. bHR lineage in the F_0_s (*n* = 13,339 genes, R = −0.196, *p* < 2e-16), which matches the prediction that a bLR-like pattern of gene expression resembles the expression associated with lower exploratory activity. Following the plotting conventions from Figure 5B, this negative correlation is illustrated using a two-sided RRHO plot. Within the RRHO, the results are ranked by t-statistic. The visible blue diagonal indicates a negative correlation between the ranked results. The correlation between bLR vs. bHR differential expression and gene expression associated with the other F_2_ behaviors can be found in Supplementary Table S6. **(B)** A pink Venn Diagram illustrates the enrichment of bLR-upregulated differentially expressed genes for nominal (*p* < 0.05) associations with bLR-like behavior in the F_2_s (i.e., gene expression correlated with decreased locomotor activity, decreased distance traveled, increased immobility, decreased % time in the open arms of the EPM, or decreased PavCA Index) (enrichment: Fisher’s exact test: OR: 3.27, *p* < 2.2e-16). **(C)** A green Venn Diagram illustrates the enrichment of bHR-upregulated differentially expressed genes for nominal (*p* < 0.05) associations with bHR-like behavior in the F_2_s (i.e., gene expression correlated with increased locomotor activity, increased distance traveled, decreased immobility, increased % time in the open arms of the EPM, or increased PavCA Index) (enrichment: Fisher’s exact test: OR 2.72, *p* = 7.14e-13). We prioritized the 192 genes that satisfied both criteria (i.e., the intersection of the Venn Diagrams) in downstream analyses as differential expression that might mediate the effect of selective breeding on behavior (111 genes upregulated in both bLRs and with bLR-like behavior, 81 genes downregulated in both bHRs and with bHR-like behavior).

Since some bHR/bLR differential expression may be due to either linkage disequilibrium with causal variants or genetic drift specific to our bred lines, we performed RNA-Seq on hippocampal tissue from a large F_2_ intercross sample (*n* = 250) to identify differential expression that continued to independently correlate with exploratory locomotion, anxiety-like behavior, and reward-related behavior. Hippocampal gene expression associated with bLR lineage resembled the expression associated with lower F_2_ LocoScore, as indicated by the negative correlation between the F_0_ bLR vs. bHR Log2FCs for all genes and the F_2_ LocoScore Log2FCs for all genes (R = −0.20, *p* < 2.2e-16, Figure 6A, Supplementary Table S6). Similarly, gene expression associated with bLR lineage partially resembled expression in F_2_s exhibiting lower exploration (distance traveled) and greater anxiety (greater time immobile, less time in the open arms) on the elevated plus maze (EPM) task (Supplementary Table S6), and greater goal-tracking behavior on the PavCA task (lower PavCA Index; Supplementary Table S6). This pattern of correlations confirmed that a portion of the hippocampal differential expression that emerged following selective breeding was related to behavioral temperament.

These correlations strengthened when focusing specifically on bHR/bLR differentially expressed genes (1,063 genes in Figures 5C–E), of which 1,045 were present in the F_2_ dataset (Supplementary Table S6). Of these genes, 111 showed both upregulation in bLR rats and at least one nominal (*p* < 0.05) association with bLR-like behavior in the F_2_s (i.e., expression correlated with decreased locomotor activity, decreased EPM distance traveled, increased EPM time immobile, decreased EPM % time in open arms, or decreased PavCA Index), a 3.27X enrichment beyond random chance (Fisher’s exact test: 95%CI: 2.61–4.08, *p* < 2.2e-16, Figure 6B), and 81 genes showed both upregulation in bHR rats and at least one nominal (*p* < 0.05) association with bHR-like expression in the F_2_s, a 2.72X enrichment beyond random chance (Fisher’s exact test: 95%CI: 2.10–3.51, *p* = 7.14e-13, Figure 6C).

However, we were unable to identify differentially expressed genes for F_2_ behavior with strong enough effects to survive false discovery rate correction (FDR < 0.10). This was also true when using a model that included sex-specific differential expression for F_2_ behaviors (sex*behavior interaction: all FDR < 0.10). This inability to detect significant differential expression related to F_2_ behavior was particularly striking because the F_2_ sample size was much larger than the sample sizes used to detect differential expression in our bred model (F_2_: *n* = 250, F_0_: *n* = 24), and this greater statistical power lead to the expected increase in the detection of more subtle differential expression related to sex (1,679 genes with FDR < 0.10, Supplementary Figure S5, full results: Supplementary Table S4).

These findings drive home the role of cumulative small, polygenic effects in generating complex behavior, and suggest a need for larger sample sizes to reliably detect these polygenic effects on gene expression in a heterogeneous population. These findings also demonstrate the utility of selective breeding in behavioral genetics: the highly divergent phenotype and minimized within-group variability made it possible to detect relevant differential expression in a much smaller size. For downstream analyses, we chose to focus on the differential expression with the strongest converging evidence supporting its potential to mediate behavioral temperament from both the selectively bred lines and F_2_ rats (the 111 genes that were upregulated in bLRs and nominally with bLR-like behavior in the F_2_s and 81 genes upregulated in bHRs and nominally with bHR-like behavior in the F_2_s).

### 3.5 Multiple genes have hippocampal differential expression consistently associated with behavioral temperament in other rat models as well as in our F_0_ and F_2_ studies

**Figure 7:**
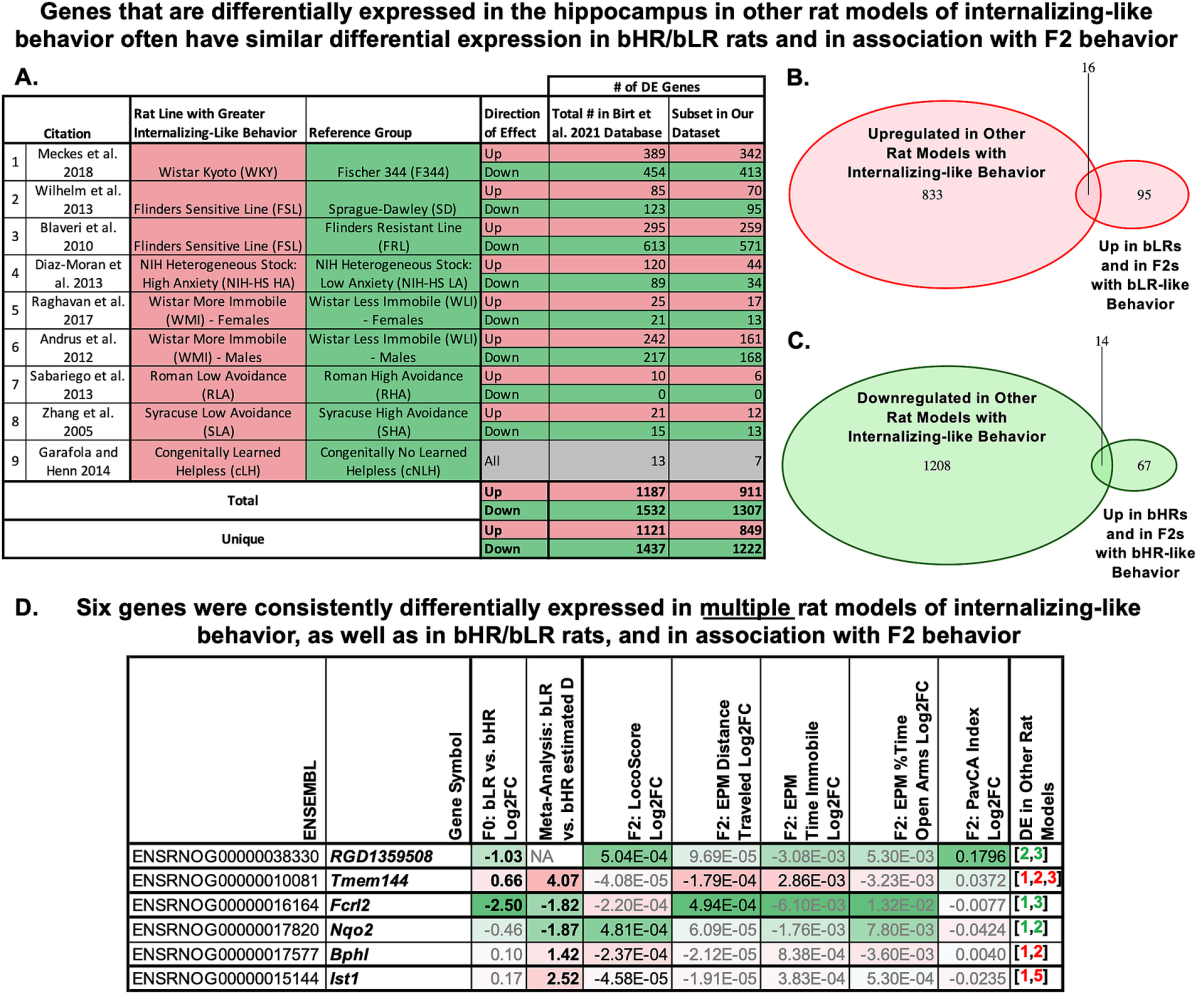
Multiple genes have hippocampal differential expression (DE) consistently associated with hereditary behavioral temperament in other rat models as well as in our F_0_ and F_2_ studies. **(A)** To perform this analysis, we compared our current results to a database of 2,581 genes that had been previously identified as differentially expressed in the hippocampus of other bLR-like and bHR-like rat models targeting hereditary behavioral traits resembling extremes on the internalizing/externalizing spectrum (database compiled in Birt et al., 2021, results from: Andrus et al., 2012; Blaveri et al., 2010; Díaz-Morán et al., 2013; Garafola and Henn, 2014; Meckes et al., 2018; Raghavan et al., 2017; Sabariego et al., 2013; Wilhelm et al., 2013; Zhang et al., 2005). The table lists the rat models characterized in the referenced publications, the number of genes up-regulated or down-regulated in association with the rat model exhibiting more internalizing-like behavior (in total, as well as the subset represented in our current datasets). **(B)** A pink Venn Diagram illustrates the enrichment of overlap between genes identified as upregulated in other rat models with internalizing-like behavior and the 111 genes that were both upregulated in bLR’s and nominally upregulated in F_2_s with bLR-like behavior in our study (16/111, enrichment OR: 2.50, Fisher’s exact test: *p* = 0.00242). **(C)** A green Venn Diagram illustrates the enrichment of overlap between genes identified as down-regulated in other rat models with internalizing-like behavior and the 81 genes that were both upregulated in bHRs and nominally upregulated in F_2_s with bHR-like behavior in our study (14/81, enrichment OR: 2.07, *p* = 0.0189). **(D)** A table overviewing the differential expression results for the six genes that were consistently differentially expressed in *multiple* rat models with internalizing-like behavior, as well as in bHR/bLR rats and in nominal association with F_2_ behavior. Within the table, genes with elevated hippocampal expression in bLRs and in association with bLR-like behavior in the F_2_s are highlighted pink, genes with elevated hippocampal expression in bHRs and in association with bHR-like behavior in the F_2_s are highlighted green. The table includes the Log2FC for bLR vs. bHR Lineage in the F_0_ dataset, the estimated effect size (d) from the late generation bLR vs. bHR RNA-Seq meta-analysis (from Birt et al., 2021), and the Log2FC for LocoScore, EPM Time Immobile, EPM Distance traveled, EPM % Time in Open Arms, and PavCA index in the F_2_ dataset (Bold = FDR < 0.1; black = *p* < 0.05). Note that the F_2_ differential expression analysis includes behavior as a continuous predictor variable, therefore the Log2FC units are defined per unit of LocoScore and not directly comparable to the Log2FC units for bred line (bLR vs. bHR). The final column provides references for similar hippocampal differential expression in other bLR-like (red) or bHR-like (green) rat models following the numbering in the table in panel **A**. Full gene names (when applicable): *Tmem144*: Transmembrane Protein 144; *Fcrl2*: Fc Receptor-like 2; *Nqo2*: N-ribosyldihydronicotinamide:quinone dehydrogenase 2; *Bphl*: biphenyl hydrolase like; *Ist1*: Factor Associated With ESCRT-III.

To determine generalizability, we compared our list of differentially expressed genes implicated in behavioral temperament by converging evidence from the bred lines and the F_2_s (identified in Figures 6B,C) to a database of 2,581 genes previously identified as differentially expressed in the hippocampus of other bLR-like and bHR-like rat models targeting hereditary behavioral traits resembling extremes on the internalizing/externalizing spectrum [database from Birt et al. (2021), summarized in Figure 7A; Andrus et al., 2012; Blaveri et al., 2010; Díaz-Morán et al., 2013; Garafola and Henn, 2014; Meckes et al., 2018; Raghavan et al., 2017; Sabariego et al., 2013; Wilhelm et al., 2013; Zhang et al., 2005]. Sixteen of 111 genes that were upregulated in bLRs and with bLR-like behavior in our study were also upregulated in other bLR-like models (Figure 7B, enrichment OR: 2.50 (95%CI: 1.37–4.29), Fisher’s exact test: *p* = 0.00242) and 14/81 genes that were upregulated in bHRs and with bHR-like behavior in our study were downregulated in other bLR-like models [Figure 7C, enrichment OR: 2.07 (95%CI: 1.07–3.74), *p* = 0.0189]. Notably, *Tmem144* had elevated hippocampal expression in three other bLR-like rat models (Figure 7D, Blaveri et al., 2010; Meckes et al., 2018; Wilhelm et al., 2013). Five other genes were differentially expressed in two other rat models (Figure 7D, *Bphl*, *Ist1*, *RGD1359508*, *Nqo2*, *Fcrl2*). We expect less than one gene (0.39) in our dataset to show this degree of convergence due to random chance (Supplementary methods).

### 3.6 Behavioral temperament is associated with genes involved in growth and proliferation, mitochondrial function, oxidative stress, and microglia

To ascribe functional trends to the differential expression associated with behavioral temperament, we performed Gene Set Enrichment Analysis using a combined score for each gene summarizing bLR-like vs. bHR-like expression across the bHR/bLR and F_2_ analyses. Sixty-three gene sets were upregulated (FDR < 0.05) with a bHR-like phenotype (i.e., in bHRs and with bHR-like F_2_ behavior; Supplementary Table S7). Nineteen of these implicated hippocampal subregions or cell types, mostly neuronal (*n* = 10), emphasizing GABA-ergic cells (*n* = 3) and dendrites (*n* = 3). The dentate gyrus was implicated (*n* = 1), epithelial cells (*n* = 4, including gene *C2cd3*) and vasculature (*n* = 3, including *Mfge8*). Fourteen gene sets were derived from previous differential expression experiments (Baker et al., 2012; Lim et al., 2021), with most related to stress or fear conditioning (*n* = 12, upregulated: *n* = 9, including gene *C2cd3*). Other implicated functions included nervous system development, proliferation, and cell fate (*n* = 13, including genes *Mfge8*, *Nqo2*, *Ucp2*, and *C2cd3*) and transcription regulation (*n* = 9, including *Ucp2*).

Thirty-seven gene sets were upregulated (FDR < 0.05) with a bLR-like phenotype (i.e., in bLRs and with bLR-like F_2_ behavior). Eleven of these implicated hippocampal subregions or cell types, especially microglia (*n* = 8, including gene *Tmem144*). Other emphasized pathways included mitochondrial function, oxidative phosphorylation, and cellular respiration (*n* = 6, including genes *Wdr93* and *Idh1*), metabolism (*n* = 5, including *Pex11a*, *Lsr*, *Ist1*, and *Idh1*), and immune response (*n* = 4). A non-directional analysis produced weaker results (12 gene sets with FDR < 0.10) highlighting similar functions (metabolism: *n* = 3, including *Pex11a*, *Lsr*, *Ist1*, *Mcee*, and *Idh1*; and microglia: *n* = 4, including *Fcrl2* and *Tmem144*). Gene sets related to a bLR-like model, Flinders Sensitive Line, were also highlighted (*n* = 3).

### 3.7 Constructing a hippocampal *cis*-eQTL database to determine which differential expression is most likely driven directly by proximal genetic variation

To identify hippocampal gene expression that might be influenced by proximal genetic variation, we integrated our current F_2_ RNA-Seq data (*n* = 245) with previous genotyping results [*n* = 4,425,349 single nucleotide polymorphisms (SNPs) (Chitre et al., 2023)] to identify 5,351 genes (eGenes) with hippocampal expression tightly correlated (FDR < 0.05) with nearby genetic variation [*cis*-eQTLs: within ±1 MB of the transcription start site (TSS)]. Using stepwise regression, we identified additional conditionally-independent *cis*-eQTLs beyond the strongest *cis*-eQTL for each eGene (Supplementary Figure S6A), distinguishing a final total of 5,937 *cis*-eQTLs representing 5,836 unique eVariants. Like previous *cis*-eQTL analyses, these eVariants were predominantly located within ±400 kB of the TSS of their respective eGene (Supplementary Figure S6B). A comparison with existing rat *cis*-eQTL databases [RatGTEx: Hong-Le et al. (2023), and other tissues in RatGTEx: https://dx.doi.org/10.17504/protocols.io.rm7vzyk92lx1/v1], Supplementary Figure S6C, indicated that most hippocampal eGenes were also significant eGenes within at least four other tissues (out of 11 tissues characterized, Supplementary Figure S6D), and confirmed that previously-identified brain ciseQTLs showed a similar direction of effect on gene expression within the hippocampus (R = 0.67–0.75, rho = 0.65–0.77, Supplementary Figure S8, S9) when there was at least a nominal (*p* < 0.05) relationship in our dataset, although many *cis*-eQTLs remained region specific. As our hippocampal *cis*-eQTL database represents a valuable resource for the interpretation of rat genomic results, we have shared it on RatGTEx.^3^

### 3.8 bHR/bLR differential expression can be predicted using the hippocampal *cis*-eQTL database

**Figure 8:**
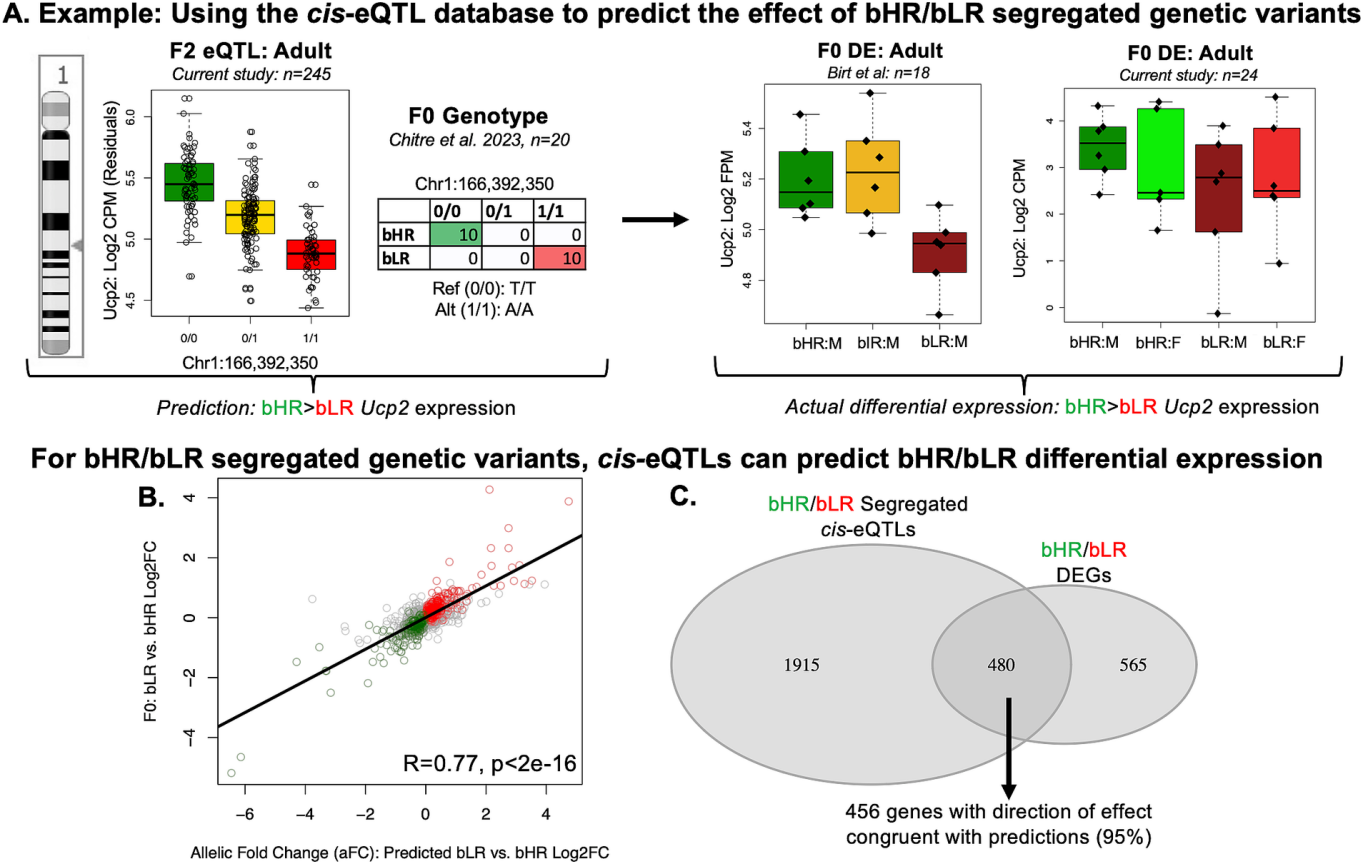
bHR/bLR differential expression (DE) related to segregated genetic variation can be successfully predicted using our hippocampal *cis*-eQTL database. Green vs. red coloring is used to indicate either bHR vs. bLR phenotype or the allele overrepresented in each respective phenotype. Gold is used to indicate heterozygotes (0/1). **(A)** To create the cis-eQTL database, we integrated our current F_2_ transcriptional profiling data (*n* = 245) with our previous whole genome sequencing results (*n* = 4,425,349 single nucleotide polymorphisms (SNPs), Chitre et al., 2023) to identify genes with hippocampal expression tightly correlated with nearby genetic variation (cis-eQTLs, FDR < 0.05). As an example, a boxplot illustrates a cis-eQTL for *Ucp2* (Uncoupling Protein 2). Within this cis-eQTL, the alternate allele for the top eVariant (Chr1:166,392,350) is associated with decreased expression of *Ucp2*. In the boxplot, genotype (x-axis) is indicated by alternate allele count, with 0/0 (two reference alleles), 0/1 (heterozygote), and 1/1 (two alternate alleles). Gene expression (y-axis: Log2 CPM) is plotted as residual expression after quality control and controlling for technical co-variates included in our differential expression model (*n* = 245). We used the cis-eQTL database to predict the effect of genetic variation that segregates the bHR/bLR lines [2,452 eVariants with partial segregation (G_st’_ > 0.27) in the F_0_ rats: *n* = 20 (Chitre et al., 2023)] on gene expression, with the direction of effect for the allelic Log2 fold change (aFC) for each eVariant assigned to reflect bLR vs. bHR allele frequency [*n* = 20 (Chitre et al., 2023)]. To illustrate this, a table shows the bHR/bLR segregation for the top eVariant (Chr1: 166,392,350) for *Ucp2*. The alternate allele (1/1) for the top eVariant is more prevalent in bLRs (red), whereas bHRs are more likely to carry the reference allele (0/0, green) (Chitre et al., 2023). Since the alternate allele was associated with decreased hippocampal *Ucp2* expression in our cis-eQTL analysis, we predict that bHRs would have greater *Ucp2* expression than bLRs. This prediction is correct when we examine our previous differential expression results from the male bHR vs. bLR rats [example boxplot from our previous F_0_ sample (Birt et al., 2021): *n* = 18, y-axis: Log2 FPM]. This prediction is also correct when we examine the differential expression results from our current F_0_ sample of male (M) and female (F) bHR and bLR rats (*n* = 24, boxplot y-axis: Log2 CPM), although the effect appears larger in males. **(B)** When considering the full sample of bHR/bLR segregated cis-eQTLs, there is a strong positive correlation between predicted bLR vs. bHR differential expression (scatterplot x-axis: bLR vs. bHR aFC) and our F_0_ differential expression results (y-axis: bLR vs. bHR Log2FC) (*n* = 2,452 cis-eQTLs, R = 0.77, *p* < 2e-16). A similar positive correlation with bLR vs. bHR meta-analysis results is shown in Supplementary Figure S10. Within the scatterplot, color indicates the subset of cis-eQTLs that were associated with differentially expressed genes upregulated in the bLRs (red) or bHRs (green) within the F_0_ differential expression study or bHR/bLR meta-analysis (Figures 4F–H) that had differential expression reflecting bHR/bLR segregation at their eVariant (*n* = 492 cis-eQTLs representing 456 eGenes). This subset is also indicated with **(C)** A Venn diagram illustrating overlap between the bHR/bLR differentially expressed genes (DEGs) (1,045 of which were present in the F_2_ dataset) with the significant eGenes identified in our hippocampal cis-eQTL database that had eVariants segregated in the bHR/bLR rats (2,395 eGenes). Out of the 480 genes satisfying both criteria, 456 (95%) showed a direction of effect in the differential expression results congruent with what would be predicted based on bHR/bLR genotype segregation.

We used our *cis*-eQTL database to predict the effect of genetic variation that segregates the bHR/bLR lines on gene expression. Many eVariants (2,452) showed at least partial bHR/bLR segregation in the F_0_ rats (*n* = 10 bHR/*n* = 10 bLR sequenced in Chitre et al., 2023), such that if all subjects from one phenotype (e.g., bHRs) had 2 reference alleles (0/0), all subjects from the other phenotype had at least 1 alternate allele (0/1); population segregation statistic G_st’_ > 0.27 (Hedrick, 2005). To predict the effect of these bHR/bLR segregated eVariants on gene expression, we calculated the allelic Log2FC (aFC) for each eVariant and assigned the direction of effect based on the allele frequency within the bLR vs. bHR F_0_ rats (Figure 8A). These predictions correlated strongly with the F_0_ differential expression results (Figures 8B,C, 2,500 eGene/eVariant combinations: R = 0.77, rho = 0.63, *p* < 2e-16) and our previous bHR/bLR late generation meta-analysis effect sizes (2,114 eGene/eVariant combinations: R = 0.52, rho = 0.61, *p* < 2e-16, Supplementary Figure S10). These results validated our hippocampal *cis*-eQTL database and confirmed that bHR/bLR differential expression of eGenes is likely driven by bHR/bLR genetic segregation.

### 3.9 *cis*-eQTLs that strongly co-localize with QTLs for behavior are predominantly located on chromosome 1

**Figure 9:**
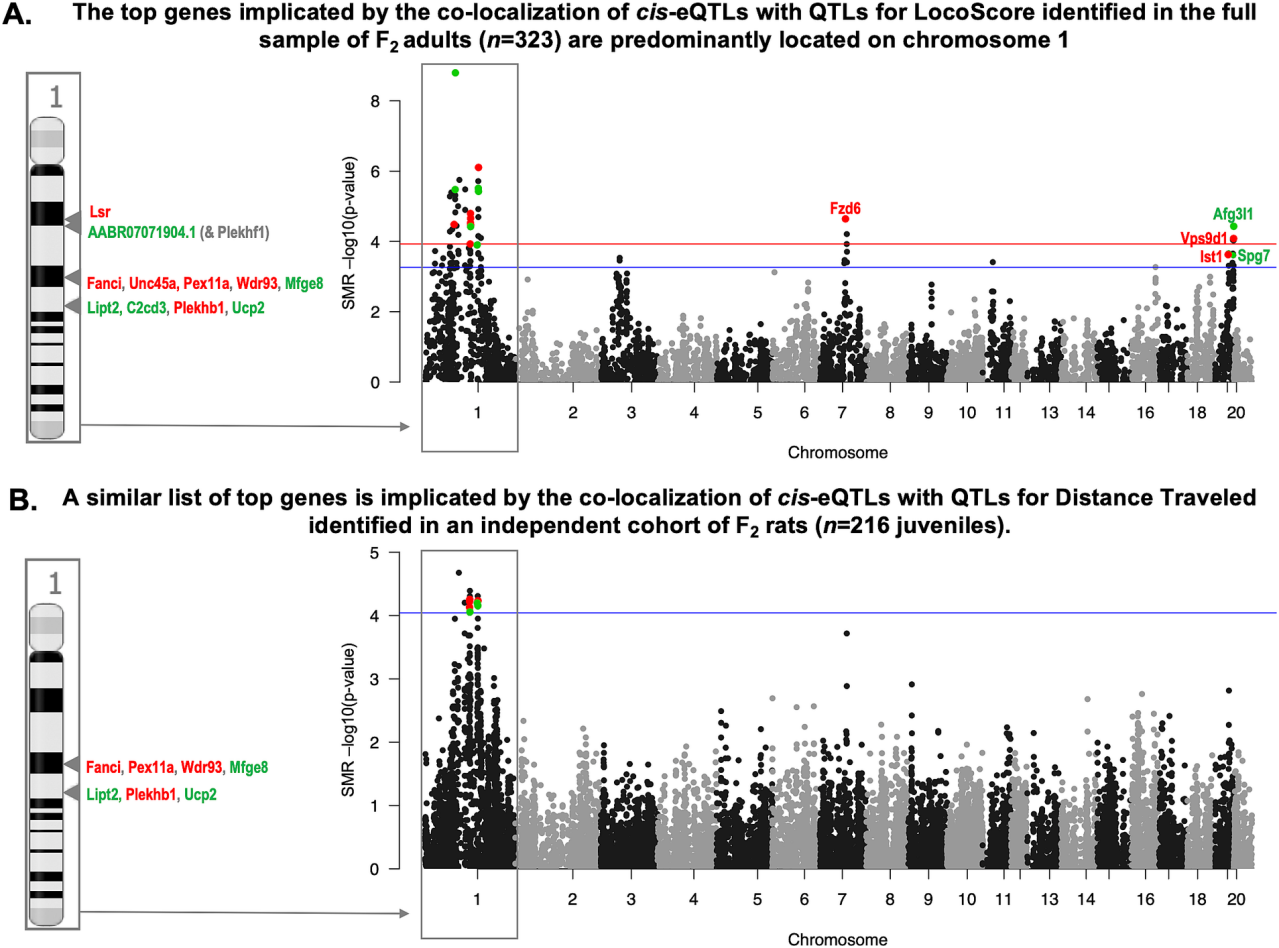
The top candidate genes for mediating the influence of genetic variation on behavioral temperament are located on chromosome 1. **(A)** A Manhattan plot shows the co-localization of hippocampal cis-eQTLs with LocoScore QTLs identified in the full sample of F_2_ adults (*n* = 323). The x-axis indicates the chromosomal location for all identified hippocampal cis-eQTLs (*n* = 5,937). Chromosomes are indicated with alternating black and grey coloring. The y-axis indicates the statistical significance [−log10(*p*-value)] for the co-localization as identified by the SMR analysis. The red line indicates FDR = 0.05 and the blue line indicates FDR = 0.10. Colored dots denote cis-eQTLs with FDR < 0.10 that meet all additional desired criteria for being the most compelling candidates for mediating the effect of selective breeding on behavior. Red is used to indicate cis-eQTLs associated with genes upregulated with bLR-like behavior (decreased LocoScore), green is used to indicate cis-eQTLs associated with genes upregulated with bHR-like behavior (increased LocoScore). For labeling the cis-eQTLs with their respective gene symbols, a side panel that zooms in on chromosome 1 is used for clarity. **(B)** A Manhattan plot shows the co-localization of cis-eQTLs with QTLs for open field distance traveled identified in an independent sample of F_2_ juveniles (*n* = 216). Notably, a similar panel of cis-eQTLs on chromosome 1 are identified as meeting all desired criteria for being the most compelling candidates for mediating the effect of selective breeding on behavior.

To identify *cis*-eQTLs that might mediate the influence of genetic variation on behavior, we determined which hippocampal *cis*-eQTLs co-localized with regions of the genome associated with bHR/bLR-like behavior (QTLs) within the larger F_2_ sample [adults: *n* = 323 adults, juveniles: *n* = 216 (Chitre et al., 2023)] using Summary Data-based Mendelian Randomization (SMR; Zhu et al., 2016). We focused on QTLs for behaviors measured in F_2_ adults that were included in our differential expression analysis (LocoScore, EPM time immobile, EPM distance traveled, EPM % time in open arms, PavCA Index), and for analogous behaviors measured in an independent sample of F_2_ juveniles (open field (OF) time immobile, OF distance traveled, OF % time in center). This analysis identified 79 *cis*-eQTLs that were co-localized with QTLs for LocoScore (FDR < 0.10), including 1 *cis*-eQTL that was also co-localized with a QTL for EPM distance traveled (FDR < 0.10), and 13 of the 14 *cis*-eQTLs that were co-localized with QTLs for OF distance traveled (FDR < 0.10). Most *cis*-eQTLs that strongly co-localized with behavioral QTLs were on chromosome 1, as expected due to the strength of the QTLs on this chromosome (Figures 9A,B).

**Figure 10:**
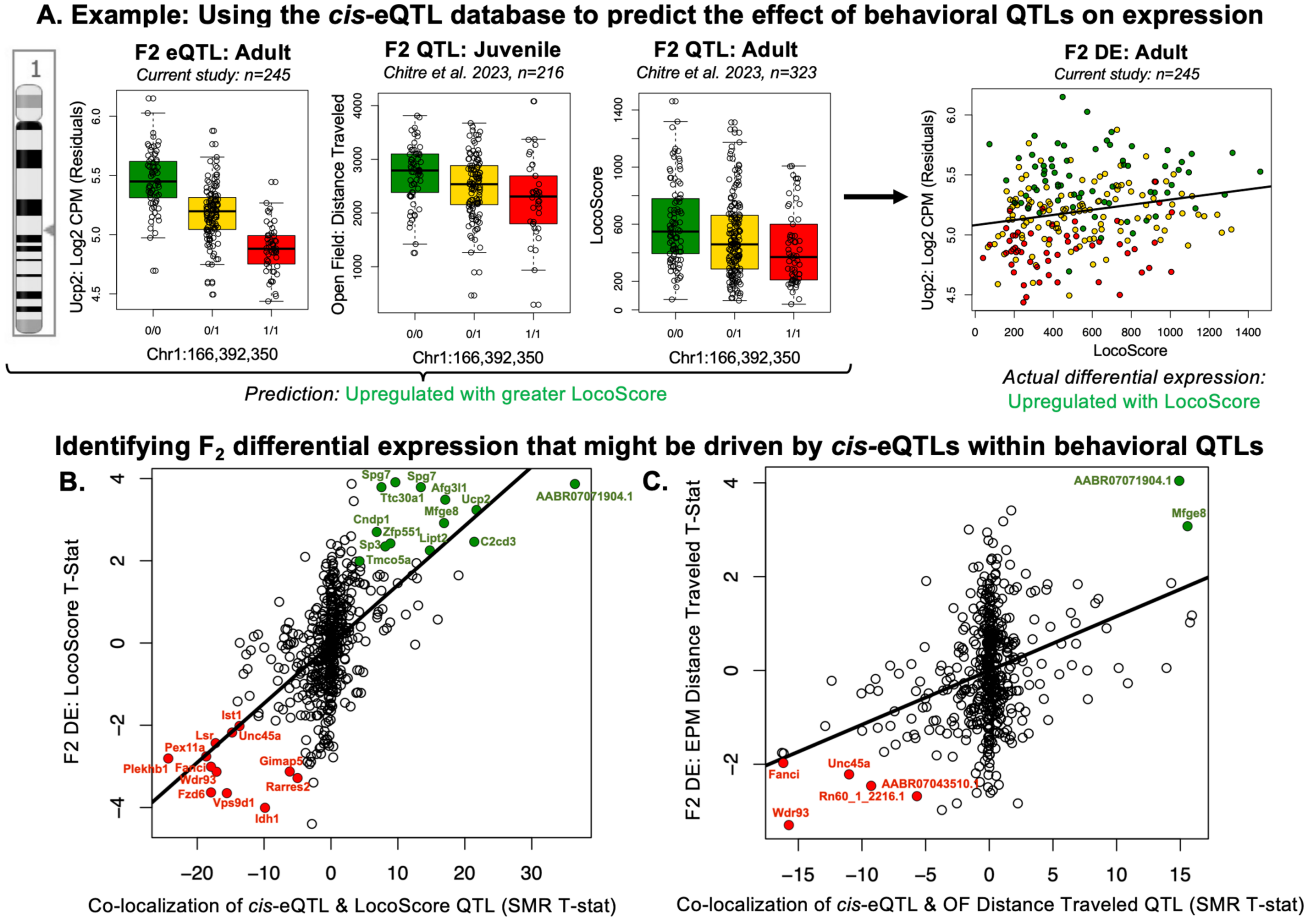
Our *cis*-eQTL database can also be used to predict the effect of genetic variation that correlates with behavior (QTLs) on gene expression. **(A)** Following the plotting conventions of Figure 8, the boxplot illustrating the cis-eQTL for *Ucp2* is shown again as an example (top eVariant: Chr1: 166,392,350), along with two boxplots illustrating the association between the alternate allele (1/1) for the top eVariant and decreased open field distance traveled in F_2_ juveniles (*n* = 216; Chitre et al., 2023) and decreased LocoScore in the full sample of F_2_ adults (*n* = 323; Chitre et al., 2023). Since the alternate allele was associated with decreased *Ucp2* expression, we predict that decreased *Ucp2* expression might also be associated with decreased distance traveled and LocoScore (i.e., positive correlation). This prediction is correct when we examine the differential expression (DE) results from the F_2_ rats (*n* = 245). The scatterplot shows that *Ucp2* was more highly expressed in hippocampus of F_2_ rats with a higher LocoScore. Similar to the eQTL plot, gene expression (Log2 CPM) is plotted as residual expression. **(B,C)** Overall, some F_2_ differential expression related to behavior can be predicted by the co-localization of cis-eQTLs with QTLs for the behavior identified within the larger F_2_ sample (adults: *n* = 323, juveniles: *n* = 216, Chitre et al., 2023). This was particularly true when considering the subset of cis-eQTLs we confirmed had differential expression in bHR/bLRs matching what would be expected based on the segregated distribution of alleles in the two lines (see Figure 8C: *n* = 492 cis-eQTLs representing 456 eGenes), but also weakly true within the full sample of cis-eQTLs (Supplementary Figures S10, S11). The strength of the co-localization of hippocampal cis-eQTLs with regions of the genome associated with bHR/bLR-like behavior (QTLs) was determined using Summary Data-based Mendelian Randomization (SMR), and the direction of effect for the relationship between gene expression and behavior was predicted as described above. **(B)** An example scatterplot shows the positive correlation between the strength of the co-localization of cis-eQTLs with the QTLs for LocoScore identified in the larger F_2_ sample (*n* = 323 adults, x-axis: SMR T-statistic), with negative values indicating a predicted negative relationship between gene expression and LocoScore and positive values indicating a positive relationship between gene expression and LocoScore and the differential expression for LocoScore (y-axis: Log2FC) (*n* = 492; R = 0.56, *p* < 2.2e-16). Color is used to indicate the subset of genes that had nominal differential expression in the F_2_s for LocoScore (*p* < 0.05) that matched the prediction based on the co-localization between their cis-eQTL and the QTL for LocoScore in the larger F_2_ sample (*p* < 0.05), with green indicating bHR-like upregulation with increased LocoScore and red indicating bLR-like upregulation with decreased LocoScore. **(C)** An example scatterplot shows the positive correlation between the strength of the co-localization of cis-eQTLs with the QTLs for open field distance traveled identified in an independent sample of F_2_ rats (*n* = 216 juveniles, x-axis: SMR T-statistic), with predicted direction of effect assigned as discussed above, and the differential expression for EPM distance traveled in the F_2_ adults (y-axis: Log2FC) (*n* = 492; R = 0.35, *p* < 1.48e-15). Coloring follows the conventions in panel **(B)**. Supplementary Figures S11, S12 contain scatterplots for other F_2_ adult and juvenile behaviors.

To narrow down our pool of top candidate genes for mediating the effect of genetic variation on behavioral temperament, we used converging information from our different samples and analyses. First, we narrowed our scope to *cis*-eQTLs that we had confirmed are segregated in bHR/bLRs with differential expression matching predictions based on the distribution of alleles in the two lines (Figure 8C; 492 cis-eQTLs representing 456 eGenes). Within this subset of *cis*-eQTLs, the strongest co-localization with QTLs tended to predict F_2_ differential expression with behavior, especially when considering the predicted direction of effect based on the relationship between genotype and behavior within the larger F_2_ sample (adults: *n* = 323 adults) and genotype and expression within the *cis*-eQTL analysis (*n* = 245) (Figure 10A). This was particularly true for LocoScore (Figure 10B, R = 0.56, *p* < 2.2e-16), but also other F_2_ adult behaviors (Supplementary Figure S11, R = 0.33–0.57, all *p* < 3.07e-14). It was also true when comparing F_2_ differential expression to SMR co-localization results with QTLs for two analogous juvenile behaviors (Figure 10C, Supplementary Figure S12, OF distance traveled: R = 0.35, *p* = 1.48e-15, OF time immobile: R = 0.26, *p* = 4.44e-09).

**Figure 11:**
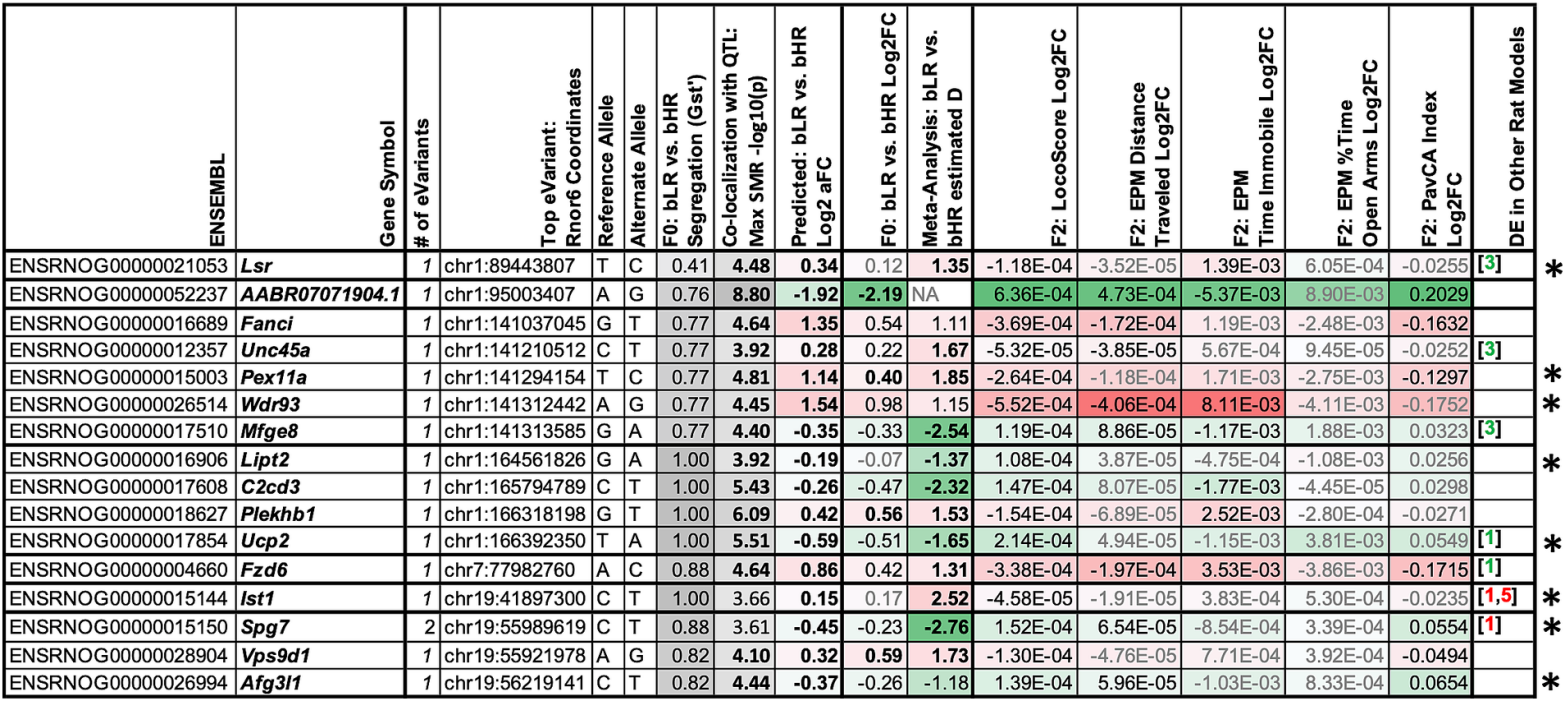
A table summarizing the converging evidence from genetic association and differential expression studies implicating 16 genes in behavioral temperament. To narrow down our pool of top candidate genes for mediating the effect of genetic variation on behavior, we used converging information from our different samples and analyses (Figure 2). We required that our top candidate genes have expression strongly related to genetic variation (cis-eQTLs) that was segregated in the bHR/bLR lines that correctly predicted bHR/bLR differential expression and co-localized with a QTL for behavior (SMR FDR < 0.10) that correctly predicted at least nominal F_2_ differential expression associated with that behavior. The summary table follows the conventions of Figure 7D, but also includes the top eVariant associated with the expression of the gene within our cis-eQTL analysis, along with its reference and alternate alleles, its separation in our bred lines [G_st’_: ranges from 0 (no segregation) to 1 (fully segregated)], its co-localization with behavioral QTLs from the full adult and juvenile F_2_ samples (maximum −log10(*p*-value) from the SMR analysis, bold = FDR < 0.05, black = FDR < 0.10), and the differential expression that is predicted due to bLR vs. bHR segregation at the eVariant (allelic Log2 fold change or Log2aFC, bold = FDR < 0.05). Genes with functions related to bioenergetics are indicated with an * and illustrated in Figure 12. Full gene names (when applicable): *Lsr:* Lipolysis Stimulated Lipoprotein Receptor; *Fanci*: FA Complementation Group I; *Unc45a:* Unc-45 Myosin Chaperone A; *Pex11a:* Peroxisomal Biogenesis Factor 11 Alpha; *Wdr93:* WD Repeat Domain 93; *Mfge8:* Milk Fat Globule EGF And Factor V/VIII Domain Containing; *Lipt2:* Lipoyl(Octanoyl) Transferase 2; *C2cd3:* C2 Domain Containing 3 Centriole Elongation Regulator; *Plekhb1:* Pleckstrin Homology Domain Containing B1; *Ucp2:* Uncoupling Protein 2; *Fzd6*: Frizzled Class Receptor 6; *Ist1:* IST1 Factor Associated With ESCRT-III; *Spg7:* SPG7 Matrix AAA Peptidase Subunit, Paraplegin; *Vps9d1:*VPS9 domain containing 1; *Afg3l1:* AFG3-like AAA ATPase 1.

**Figure 12:**
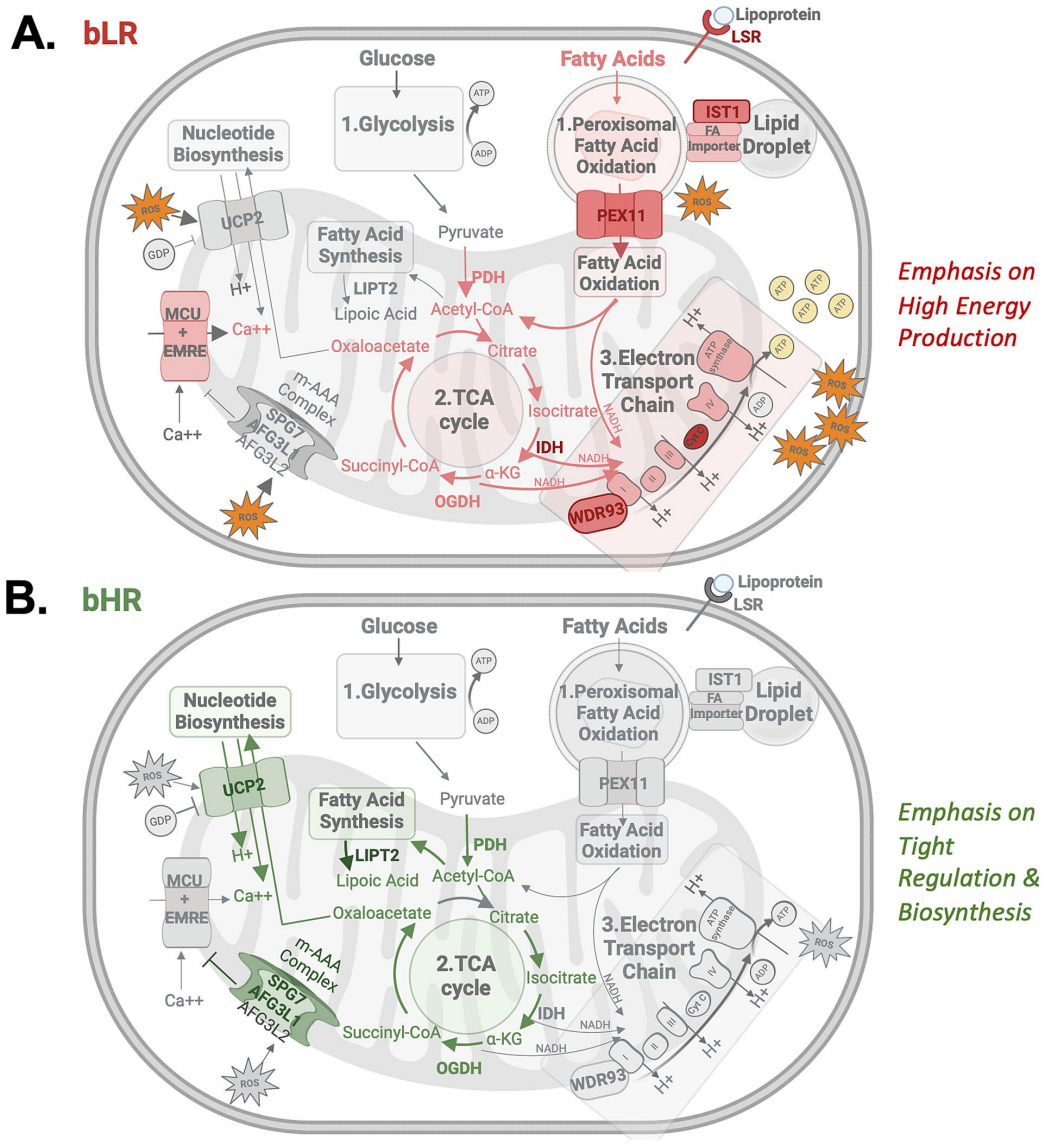
The differentially expressed genes implicated as top candidates for mediating the effect of selective breeding on behavior are often regulators of bioenergetic function. Red/pink indicates that a function is likely to be increased in bLR-like animals, green indicates increased function in bHR-like animals. 1. In the brain, energy is primarily released from glucose within a series of biochemical reactions starting with glycolysis (Sobieski et al., 2017), but it can also be released from other energy sources, such as fatty acid oxidation (Nsiah-Sefaa and McKenzie, 2016). 2. Metabolites from these processes are fed into the tricarboxylic acid (TCA) cycle within the mitochondrial matrix, which generates electron donors (NADH, FADH2). 3. Electron donors feed into the electron transport chain, which moves protons across the inner mitochondrial membrane to produce a gradient capable of driving energy output (adenosine triphosphate: ATP). This process produces reactive oxygen species (ROS) as a byproduct (Zeng et al., 2020). **(A)** bLR-like animals have a pattern of upregulated expression suggesting elevated oxidative phosphorylation, but potentially also reduced sensitivity to cellular need in a manner leading to excessive ROS production and neuroimmune activation under conditions of elevated activity such as stress. This conclusion is supported by: **(I)** previous evidence of elevated oxidative phosphorylation, including elevated activity within the electron transport chain (McCoy et al., 2019), **(II)** Upregulation of multiple gatekeepers of fatty acid oxidation, which is a form of energy production that can release twice as much energy as glucose metabolism (Schönfeld and Reiser, 2013). These gatekeepers include lipolysis stimulated lipoprotein receptor (*Lsr*), which uptakes lipoproteins into the cell, IST1 factor associated with ESCRT-III (*Ist1*), which facilitates fatty acid trafficking into peroxisomes to begin fatty acid oxidation (Chang et al., 2019), and peroxisomal biogenesis factor 11 alpha (*Pex11a*), which encodes a fatty acid oxidation rate-limiting channel that allows lipids and fatty acid metabolites to pass from peroxisomes into mitochondria (Mattiazzi Ušaj et al., 2015; Mindthoff et al., 2016; Renne and Ernst, 2023). **(III)** Upregulation of *Idh1*, encoding the isocitrate dehydrogenase 1 enzyme in the TCA cycle, which is the most important producer of the electron donor NADH in the brain (Gherardi et al., 2020; Molenaar et al., 2014), **(IV)** Upregulation of WD repeat domain 93 (Wdr93), which is theorized to be an accessory subunit to Complex 1 in the mitochondrial electron transport chain, increasing ATP production (GeneCards, n.d.; InterPro, n.d.; Meyer et al., 2009). **(V)** Down-regulated expression of subunits of the m-AAA (ATPases Associated with a variety of cellular Activities) complex [Spastic paraplegia type 7 (*Spg7*), AFG3-like protein 1 (*Afg3l1*)]. Decreased m-AAA complex function causes constitutive activity of the mitochondrial calcium uniporter (MCU) (König et al., 2016; Patron et al., 2018). Elevated mitochondrial calcium influx activates enzymes within the TCA cycle (PDH, IDH, and OGDH) (Gherardi et al., 2020), and, if it becomes excessive, triggers ROS production and apoptosis (Patron et al., 2018). **(B)** bHR-like animals have a pattern of upregulated expression suggesting that energy production is kept under tight regulation, potentially limiting oxidative phosphorylation but allowing for greater biosynthesis. These findings include **(I)** Upregulated Uncoupling Protein 2 (*Ucp2*), encoding a mitochondrial transporter which promotes homeostasis and decreased ROS production by decreasing the mitochondrial proton gradient, exporting the rate-limiting substrate for the TCA cycle (oxaloacetate), and regulating calcium influx (Ardalan et al., 2022; Berardi and Chou, 2014; Hass and Barnstable, 2021; Keita et al., 2007; Koshenov et al., 2020; Vozza et al., 2014); **(II)** Upregulated expression related to m-AAA complex function (*Spg7*, *Afg3l1*), which ensures that mitochondrial protein availability, including an essential regulator (EMRE) of the MCU, does not exceed cellular need. **(III)** Upregulated lipoyl(octanoyl) transferase 2 (*Lipt2*), which senses the input to the TCA cycle (Acetyl-CoA) and stimulates TCA cycle enzyme activity accordingly via the mitochondrial fatty acid synthesis pathway (Habarou et al., 2017; Nowinski et al., 2020; Solmonson and DeBerardinis, 2018).

The most compelling candidates for mediating the effect of genetic variation on behavioral temperament should have expression strongly related to genetic variation (*cis*-eQTLs) that is segregated in bHR/bLR, correctly predicts bHR/bLR differential expression, and co-localizes with a QTL for behavior that correctly predicts F_2_ differential expression associated with that behavior (Figure 2). Among the SMR results, 16 of the 80 genes surviving FDR correction (FDR < 0.10) met all these criteria (Figure 11, examples: Supplementary Figures S13–S16). By conservative estimate (Supplementary methods), one gene or less in our dataset is expected to show this degree of convergence due to random chance. These 16 genes were clustered within seven genomic regions on chromosomes 1, 7, and 19, suggesting that there still remained some false discovery due to linkage disequilibrium (Figures 9A,B). That said, when cross-referencing with functional annotation, eight of these candidate genes—representing five of the identified regions—were clearly related to mitochondrial function and bioenergetics (Figures 11, 12), hinting at the relevant genes in each region.

## 4 Discussion

Using selectively-bred rats with extreme, stable differences in behavior (bHRs, bLRs) and a large cohort of their intercross progeny (F_2_s), we identified genes and functional pathways that are likely to contribute to behavioral temperament. This was achieved by triangulating behavioral, functional genomics, and genetic data. Behaviors that diverged in the bHR/bLR lines, including anxiety-like and reward-related behavior (Birt et al., 2021; Flagel et al., 2010; Turner et al., 2017), remained correlated with exploratory locomotion in our heterogeneous F_2_ sample, allowing us to investigate their shared etiology. The extreme behavioral phenotypes produced by our selective breeding paradigm were accompanied by robust differential expression in the hippocampus of both sexes, bolstering results from our previous male-only analyses (Birt et al., 2021). Moreover, hippocampal gene expression related to bHR/bLR lineage predicted gene expression related to behavior, including exploratory locomotion and anxiety, in our larger cohort (*n* = 250) of F_2_ intercross rats. Six genes showed consistent differential expression with behavioral phenotype in bHR/bLR and F_2_ intercross rats, as well as in multiple other rat models targeting similar behavior.

Selective breeding should produce an enrichment of genetic alleles influencing the phenotype under selection. To determine which differential expression might directly mediate the effect of selective breeding on behavioral temperament, we identified hippocampal expression that was strongly correlated with genetic variation in the F_2_s (*cis*-eQTLs). This *cis*-eQTL database allowed us to accurately predict differential expression related to bHR/bLR genetic segregation. We also identified gene expression associated with F_2_ behavior that matched what would be expected due to the co-localization of *cis*-eQTLs with genetic loci associated with behavior (QTLs) in the larger F_2_ cohort (adults and juveniles) (Chitre et al., 2023). This converging evidence highlighted 16 genes within 7 genomic regions on chromosomes 1, 7, and 19 as strong candidates for mediating the effect of selective breeding on behavioral temperament.

### 4.1 Functional patterns: bioenergetic regulation of hippocampal function

Among these 16 top candidate genes, eight are directly involved in bioenergetics (Figure 12). The differential expression results overall similarly showed upregulation in gene sets related to mitochondria, oxidative phosphorylation, and metabolism in bLR-like vs. bHR-like animals. These findings complement previous evidence that adult bLRs have elevated oxidative phosphorylation in the hippocampus, as indicated by increased mitochondrial oxygen consumption and elevated electron transport chain activity (McCoy et al., 2019). Since the expression of our top candidate genes was strongly correlated with genetic variation tied to behavioral phenotype in both bHR/bLR and F_2_ samples, our results imply that variation in energy production may mediate the effect of heredity on temperament and provide insight into the responsible mechanisms.

In particular, our results suggest that bLR-like animals have enhanced fatty acid oxidation, which is a pathway that is particularly important during times of high energy usage (Nsiah-Sefaa and McKenzie, 2016) because it can release twice as much energy as glucose metabolism (Schönfeld and Reiser, 2013). bLR-like animals had upregulation of multiple fatty acid oxidation gatekeepers [*Lsr*, *Ist1*, and *Pex11a* (Chang et al., 2019; Mattiazzi Ušaj et al., 2015; Mindthoff et al., 2016; Renne and Ernst, 2023)]. Downstream, there was also upregulation that could facilitate the tricarboxylic acid (TCA) cycle (*Idh1*) (Gherardi et al., 2020) and electron transport chain (*Wdr93*) (GeneCards, n.d.; InterPro, n.d.; Meyer et al., 2009) to increase energy production. These findings have widespread functional implications, as the brain consumes disproportionate energy to maintain neurotransmission and synaptic repolarization (van Rensburg et al., 2022), especially during times of heightened activity, such as stress (Zalachoras et al., 2020).

In contrast, energy production in bHR-like animals may be kept under tight regulation by upregulation of *Spg7*, *Afg3l1*, *Ucp2*, and *Lipt2*. *Ucp2* encodes a mitochondrial transporter and anion carrier that promotes homeostasis by serving as a metabolic switch, decreasing TCA cycle function (Vozza et al., 2014) and mitochondrial proton gradient (Ardalan et al., 2022; Berardi and Chou, 2014; Hass and Barnstable, 2021; Keita et al., 2007). *Lipt2* plays a similar feedback role, coupling TCA cycle enzyme activity to its input via the mitochondrial fatty acid synthesis pathway (Habarou et al., 2017; Nowinski et al., 2020; Solmonson and DeBerardinis, 2018). *Spg7* and *Afg3l1* encode subunits of the m-AAA complex, which tailor mitochondrial protein levels to cellular need (Opalińska and Jańska, 2018). Moreover, *Ucp2*, *Spg7* and *Afg3l* all regulate mitochondrial calcium intake (König et al., 2016; Koshenov et al., 2020; Patron et al., 2018), which couples energy production to synaptic activity by stimulating TCA cycle enzymes (Gherardi et al., 2020; Stoler et al., 2022). As discussed below, this tight regulation may limit oxidative phosphorylation under some conditions, but also reduce reactive oxygen species production and allow for greater biosynthesis.

### 4.2 Bioenergetics and behavior

Our results bolster growing evidence that bioenergetic genes and pathways regulate behaviors like exploratory activity, anxiety, and reward learning. The mitochondrial m-AAA complex, fatty acid oxidation pathway, and fatty acid synthesis feedback pathway are all critical for movement and motor activity in animals and humans (Bernardinelli et al., 2017; Martinelli et al., 2009; Murru et al., 2019; Nsiah-Sefaa and McKenzie, 2016; Patron et al., 2018), with severe, pathogenic mutations in *Spg7*, *Afg3l1*, and *Ist1* producing hereditary paraplegia and ataxia (Lallemant-Dudek and Durr, 2021; Nsiah-Sefaa and McKenzie, 2016; Schüle and Schöls, 2011), sometimes with altered cognition, executive function, and social/emotional function (Hedera et al., 2002; Lupo et al., 2020; Ringman et al., 2020; Schüle and Schöls, 2011; Zhang et al., 2017). Logically, more subtle changes within these pathways could alter exploratory activity.

Energy production is also theorized to critically modulate the energy-demanding circuitry necessary for behavioral inhibition (Killeen et al., 2013; Russell et al., 2006), and some of our candidate bioenergetic genes are broadly implicated in behavioral temperament. *Ucp2* knock-out animals consistently demonstrate bLR-like behaviors, including decreased exploration, and anxiety- and depressive-like behaviors, especially following stress (Andrews et al., 2006; Du et al., 2016; Gimsa et al., 2011; Hermes et al., 2016; Sun et al., 2011; Wang et al., 2014; Yasumoto et al., 2021). Human GWAS also link *UCP2*, *SPG7*, and *WDR93* to the stress response, psychiatric disorders, externalizing behavior, and substance use disorders (Karlsson Linnér et al., 2021; Li et al., 2022; Orhan et al., 2012; Pehlivan et al., 2020; Rimpelä et al., 2019; Russell et al., 2020; Saunders et al., 2022; Yasuno et al., 2007).

Metabolic differences have been observed in humans and animal models with anxiety and internalizing-like behavior (Ait Tayeb et al., 2023; Filiou et al., 2011, 2014; Filiou and Sandi, 2019; Liu et al., 2022) and hyperactivity and externalizing-like behavior (Chang et al., 2018; Dimatelis et al., 2015; Dupuy et al., 2021; Zametkin et al., 1990). Our findings suggest that genetic vulnerability may contribute to these metabolic differences, bolstering support for metabolic interventions in psychiatry (e.g., Chang et al., 2018; Danan et al., 2022; Filiou and Sandi, 2019; Liu et al., 2022). That said, the evidence linking energy production to internalizing-like vs. externalizing-like behavior is inconsistent across measurements and models, suggesting that the critical vulnerability may lie downstream in bioenergetically regulated functions like apoptosis, oxidative stress, and biogenesis (Filiou and Sandi, 2019). We have evidence supporting each of these possibilities.

### 4.3 Bioenergetics: role in reactive oxygen species production

During fatty acid oxidation and oxidative phosphorylation, reactive oxygen species are produced as a byproduct (Schönfeld and Reiser, 2021; van Rensburg et al., 2022). Both energy production and reactive oxygen species increase with elevated synaptic activity and environmental stress (Salim, 2017; van Rensburg et al., 2022; Zalachoras et al., 2020). Thus, many metabolic genes are regulators of oxidative stress, with upregulation in bLR-like animals linked to greater oxidative stress and upregulation in bHR-like animals sometimes appearing protective [e.g., *Ucp2*, *Spg7/Afg3l1*, *Pex11a* (Arsenijevic et al., 2000; Atorino et al., 2003; Du et al., 2016; Gimsa et al., 2011; Hass and Barnstable, 2021; Rodríguez-Serrano et al., 2016)]. bHR-like animals also had upregulation of protective *Mfge8* (Liu et al., 2014) and upregulation of *Nqo2*, which can enhance reactive oxygen species production or reduce oxidative stress (Janda et al., 2015; Rashid et al., 2021; Vella et al., 2005) in a manner important for encoding novelty in hippocampal interneurons (Gould et al., 2020) and potentially stress-related disorders (Bainomugisa et al., 2021).

These results bolster evidence that natural and genetically-selected variation in anxiety is consistently associated with markers of oxidative damage in animals and humans (Filiou and Sandi, 2019). Reactive oxygen species are also implicated in the development of anxiety and depressive-like behavior following chronic stress (Schiavone and Trabace, 2016; van Rensburg et al., 2022). The hippocampus is particularly vulnerable to oxidative stress (Salim, 2017) and accumulating evidence implicates oxidative stress in psychiatric disorders, including internalizing disorders and comorbid substance abuse (Bouayed et al., 2009; Cecerska-Heryć et al., 2022; Hovatta et al., 2010; Schiavone and Trabace, 2016; Tobore, 2019; van Rensburg et al., 2022; Zalachoras et al., 2020).

### 4.4 Bioenergetics: role in neuroimmune activation

Gene sets related to immune activation and microglia were upregulated in bLR-like animals. This upregulation may be driven by bLR/bHR bioenergetic differences: both the ATP and reactive oxygen species produced by fatty acid oxidation and oxidative phosphorylation can cause microglial activation (Illes et al., 2020; Rojo et al., 2014) and promote microglial release of pro-inflammatory factors (Guevara et al., 2020; Guo et al., 2022). Notably, two of the top candidates upregulated in bHR-like animals, *Ucp2* and *Mfge8*, are also master regulators of microglial activation, promoting an anti-inflammatory and pro-repair state (De Simone et al., 2015; Fang and Zhang, 2021; Gao et al., 2021). Both *Mfge8* and *Ucp2* encourage microglial engulfment of damaged cells and unwanted synapses. Disrupting this process causes hippocampal dysfunction, inflammation, anxiety-like behavior, insomnia, and depressive-like behavior (Choudhury et al., 2022; Fuller and Van Eldik, 2008; Yasumoto et al., 2021; Yi, 2016), mirroring a bLR-like behavioral phenotype. *Fcrl2* was also upregulated in bHR-like animals and is likely abundant in microglia, dampening immune responses (Hammond et al., 2019; Matos et al., 2020). In contrast, *Tmem144* was upregulated in bLR-like animals in our study and three others (Blaveri et al., 2010; Meckes et al., 2018; Wilhelm et al., 2013) and is highly expressed in microglia during development (Cao et al., 2020; La Manno et al., 2016), but with unknown function.

These findings complement previous findings that bLR microglia exhibit an “intermediate activation” hyper-ramified morphology (Maras et al., 2022) resembling that observed following chronic stress (McEwen and Akil, 2020), when reactive oxygen species and microglial activation are critical for the development of anxiety-like behavior (Guevara et al., 2020; Lehmann et al., 2019). Moreover, inhibiting microglial activity reduced bLR-like behavior (Maras et al., 2022). Microglial activation has also been implicated in affective and substance use-related behaviors (Choudhury et al., 2022; Northcutt et al., 2015).

Neuroimmune activation could also be caused by mitochondrial regulation of apoptosis and cell death. Excessive mitochondrial calcium intake, decreased m-AAA complex function, decreased *Spg7*, and decreased *Lipt2* can all trigger the mitochondrial membrane potential collapse that drives apoptosis (Bernardinelli et al., 2017; Patron et al., 2018; Shanmughapriya et al., 2015). m-AAA complex deficiencies can also cause dysfunctional mitochondrial protein synthesis, respiration, transport, and fragmentation (Patron et al., 2018) and are linked to neurodegeneration (König et al., 2016; Patron et al., 2018) whereas *Ucp2* is considered neuroprotective (Hass and Barnstable, 2016; Kumar et al., 2022). Therefore, down-regulation of *Spg7*, *Afg3l1*, *Lipt2*, and *Ucp2* in bLR-like animals might increase risk for cell loss and neuroimmune activation, especially after periods of intense neuronal activity, such as occurs during stress (Zalachoras et al., 2020).

### 4.5 Bioenergetics: role in growth

bHR/bLR bioenergetic differences may also contribute to the upregulation of gene sets related to nervous system development and proliferation in bHR-like animals. Both energy availability and use exert control over proliferation, cell differentiation, and growth-related processes, and biosynthesis using glucose-derived products directly competes with oxidative phosphorylation for essential substrates (Beckervordersandforth, 2017). Therefore, many of the candidate metabolic genes also influence proliferation and growth (e.g., *Ucp2*, *Lsr*, *Lipt2*: Esteves et al., 2014; Pecqueur et al., 2008; Takahashi et al., 2021; Wang et al., 2023; Zhang and Ma, 2021). Other top candidates regulate growth-related processes, including *Mfge8* and *Fzd6* (Wang et al., 2012; Yli-Karjanmaa et al., 2019; Zhou et al., 2018). *Fzd6* has also been linked to anxiety and depressive-like behavior (Sani et al., 2012; Voleti et al., 2012). These results are noteworthy due to known bHR/bLR differences in neurogenesis, proliferation, and growth factor response (Birt et al., 2021; Perez et al., 2009; Turner et al., 2019), and extensive literature implicating both hippocampal atrophy in internalizing disorders and growth-related processes in antidepressant function (Duman and Monteggia, 2006).

### 4.6 Sex differences

A vast literature exists documenting sex differences in anxiety and reward-related behaviors and circuitry (Bangasser and Cuarenta, 2021; Becker and Koob, 2016). Our study was not specifically designed to study these differences, but sex differences were observed for several behaviors, some of which have appeared consistently over many generations of bHR/bLR rats (anxiety-like behavior: Hebda-Bauer et al., 2017) or in previous studies. For example, more females than males demonstrated sign-tracking vs. goal-tracking PavCA behavior (Hughson et al., 2019; Pitchers et al., 2015), reflecting sex differences in reward processing (Davis et al., 2008; Flagel et al., 2011). Other sex differences may represent batch effects (e.g., LocoScore), as males and females were by necessity tested separately.

Our hippocampal differential expression analyses could shed light on these sex differences. Beyond the x- and y-chromosomes, the effect sizes for sex differences in hippocampal expression tended to be small, making them difficult to measure in the F_0_ sample, but in the larger F_2_ sample 1,679 genes had sex differences that survived false discovery rate correction (FDR < 0.10). We did not find evidence that sex modulated the relationship between gene expression and bHR/bLR phenotype or F_2_ behavior, but there was a notable overlap (10%) of the genes with sex differences in expression in either the F_0_ or F_2_ datasets with genes with bHR/bLR differential expression (Supplementary Table S4). In future studies, these genes may be excellent candidates for mediating sex differences in behavior.

### 4.7 Region specificity

We focused on a single brain region because of the need to generate a large sample size (*n* = 250) from a heterogeneous F2 population, however, each of the measured behaviors depends on the activity of broader brain circuitry. Can our hippocampal results provide insight into the functioning of other brain regions? To address this question, we ran an exploratory analysis comparing bHR/bLR hippocampal differential expression to the pattern of differential expression in other brain regions in previous transcriptional profiling datasets from bHR/bLR adults, including the amygdala (Cohen et al., 2015, 2017; McCoy et al., 2017), dorsal raphe (Cohen et al., 2017), and unpublished data from the cortex and hypothalamus. These comparisons suggest that at least some of the bHR/bLR differential expression that we identified in the hippocampus may also be present in other brain regions, whereas other differential expression may be hippocampal specific. Our ongoing studies using spatial transcriptomics and fluorescent *in situ* hybridization (FISH) should provide further insight into bHR/bLR differential expression in other brain regions, as well as illuminate the specificity of our findings to particular hippocampal cell types and subregions. We also have ongoing work characterizing bHR/bLR gene expression and chromatin accessibility in the nucleus accumbens, another region noted for its role in sensation-seeking, reward processing, and addiction.

The behaviors quantified in our F_2_ rats did not encompass all hippocampal-dependent behaviors that differ in the two lines. Notably, bHR and bLR rats show differences in contextual fear conditioning (Prater et al., 2017; Widman et al., 2019) that could be influenced by many of the pathways implicated in our results, including mitochondrial function, oxidative stress, microglial function, and neurogenesis (Gao et al., 2018; Olsen et al., 2013; Villasana et al., 2016; Yu et al., 2022). Future work following up on these findings could provide insight into the heritable contributions underlying internalizing disorders like post-traumatic stress disorder (Banerjee et al., 2017).

### 4.8 Alternative genetic mechanisms

One limitation of our approach is that loci which influence behavior may still be part of haplotypes that include multiple eQTLs, despite the added resolution provided by a F_0_-F_1_-F_2_ cross. We have addressed this limitation by integrating individual genes into higher order biological concepts. This approach improves the translatability of our results and should be robust to the presence of some false positives. That said, we may be missing genetic variation contributing to our phenotype by only focusing on single nucleotide variants that could mediate effects on behavior via basal hippocampal gene expression levels. For example, one of the most compelling candidates that we identified was *AABR07071904.1*, with a *cis*-eQTL near the strongest LocoScore QTL peak (Chitre et al., 2023). According to genome assembly Rnor6 (Ensembl v103), *AABR07071904.1* generates long non-coding RNA, but in mRatBN7.2 (Ensembl v106) the gene was retired, potentially mapping to *Zfp939-201*. In either form, it could play some important regulatory role, but it is noteworthy that the implicated *cis*-eQTL is in linkage disequilibrium with a missense coding variant for *Plekhf1* (Supplementary Figure S13, Chitre et al., 2023). *Plekhf1* was not differentially expressed in our study, but has been linked to stress and mood (Chitre et al., 2023). Future work will explore other mechanisms that may contribute to our phenotype, including coding variants, structural variants, epigenetic modifications, epistatic interactions, and context-dependent activity.

## 5 Conclusion: the power of integrative genomics methods for studying behavior

In conclusion, our study illustrates the power and utility of selective breeding in behavioral neuroscience: by maximizing genetic segregation relevant to our behavioral phenotype, we produced highly divergent behavior and minimized within-group variability, making it possible to detect robust, reproducible differential expression in a sample size (*n* = 24) akin to what is feasible for other neuroscience methods, including neurophysiology, cell-level labeling and imaging methods, single cell RNA-Seq, and spatial transcriptomics. In contrast, we discovered that our large F_2_ sample (*n* = 250) was still underpowered to reliably detect the smaller, polygenic effects on gene expression driving complex behavior in a heterogeneous population, even though the overall gene expression patterns associated with F_2_ behavior echoed the differential expression identified in our bred lines, supporting their relevance for the phenotype. However, by integrating our F_2_ functional genomics data with genotyping data from our previous genetic study (Chitre et al., 2023), we could detect hippocampal gene expression closely tied to proximal genetic variation (*cis*-eQTLs), allowing us to identify bHR/bLR segregated eVariants that were both predictive of bHR/bLR differential expression and co-localized with loci implicated in behavioral phenotype (QTLs). These integrative methods converged upon a set of bioenergetic-related genes that are strong candidates for mediating the influence of selective breeding on temperament and related behavior, including exploratory locomotion, anxiety, and reward learning. These bioenergetic genes are important for regulating many of the pathways implicated in our differential expression results, including oxidative stress, microglial activation, and growth-related processes in the hippocampus, each of which may be important contributors to behavioral temperament, thereby modulating vulnerability to psychiatric and addictive disorders. Therefore, altogether, our study highlights the power of integrating genetic and gene expression data to strengthen discovery-based approaches for revealing novel mechanisms underlying the neurobiology of behavior.

## Data Availability

Following MINSEQE reporting guidelines, all behavioral data, metadata, raw and processed sequencing data have been made available on NCBI Gene Expression Omnibus (GEO; https://www.ncbi.nlm.nih.gov/geo/ accession numbers: GSE225744 (F0), GSE225746 (F2), GSE286181 (cortex, hypothalamus)).

## Footnotes

1 Munro, D., Wang, T., Chitre, A. S., Polesskaya, O., Ehsan, N., Gao, J., et al. (2022). The regulatory landscape of multiple brain regions in outbred heterogeneous stock rats. *Nucleic Acids Res*. 50, 10882–10895. doi: 10.1093/nar/gkac912 (unpublished).

2
https://cran.r-project.org/web/packages/vcfR/vcfR.pdf

3
https://ratgtex.org/download/study-data/#HPC_F2
