# Correction: Bioenergetic-related gene expression in the hippocampus predicts internalizing vs. externalizing behavior in an animal model of temperament

**DOI:** 10.3389/fnmol.2026.1784625

**Published:** 2026-02-11

**Authors:** Elaine K. Hebda-Bauer, Megan H. Hagenauer, Daniel B. Munro, Peter Blandino Jr., Fan Meng, Keiko Arakawa, John D. H. Stead, Apurva S. Chitre, A. Bilge Ozel, Pejman Mohammadi, Stanley J. Watson Jr., Shelly B. Flagel, Jun Li, Abraham A. Palmer, Huda Akil

**Affiliations:** 1 Michigan Neuroscience Institute, University of Michigan, Ann Arbor, MI, United States; 2 Department of Psychiatry, University of California San Diego, La Jolla, CA, United States; 3 Seattle Children's Research Institute, University of Washington, Seattle, WA, United States; 4 Department of Neuroscience, Carleton University, Ottawa, ON, Canada; 5 Department of Human Genetics, University of Michigan, Ann Arbor, MI, United States; 6 Department of Pediatrics, University of Washington School of Medicine, Seattle, WA, United States; 7 Institute for Genomic Medicine, University of California San Diego, La Jolla, CA, United States

**Keywords:** temperament, hippocampus, RNA-Seq, locomotor activity, anxiety, energy metabolism, eQTL

In the published article, Supplemental Material files Table S3, Table S4, Table S5 and Table S7 were erroneously omitted. The file(s) have now been published, and the Supplementary Material files have been corrected to reflect this change.

The original version of this article has been updated.

